# Fatty acids, polyphenols and volatiles as predictive biomarkers of cold-pressed oil stability

**DOI:** 10.1016/j.fochx.2026.103663

**Published:** 2026-02-13

**Authors:** Tobias Pointner, Philipp Steinlechner, Marc Pignitter

**Affiliations:** a Department of Physiological Chemistry, Faculty of Chemistry, University of Vienna, 1090 Vienna, Austria; b Vienna Doctoral School in Chemistry (DoSChem), University of Vienna, Währinger Str. 42, 1090 Vienna, Austria

**Keywords:** Cold-pressed oils, Oxidative stability, Biomarker identification, Fatty acids, Polyphenols, Secondary lipid oxidation products

## Abstract

Cold-pressed vegetable oils are rich in PUFAs and bioactive polyphenols but are highly susceptible to oxidative deterioration. Six cold-pressed oils (black cumin, canola, sunflower, high-oleic sunflower, linseed and hempseed oil) from three Austrian suppliers each were stored under household-relevant conditions over six months and analyzed by GC-FID, LC-MS/MS and SPME-GC–MS. Biomarker identification employed Least Absolute Shrinkage and Selection Operator (LASSO), Random Forest regression (RFR) and correlation analysis. Fatty acid degradation, particularly of linoleic and α-linolenic acid, was the main driver of oxidation, while volatile aldehydes such as hexanal, 2,4-decadienal, and (E)-2-nonenal served as key secondary markers. Polyphenols exhibited pronounced, oil-type-dependent depletion during storage, with phenolic acids generally declining earlier than flavonoids and lignans, supporting their use as early-stage stability markers. This is the first study to systematically identify oil-type-specific deterioration biomarkers using an integrated, machine-learning-based multi-marker approach, demonstrating that oxidative stability in cold-pressed oils is best characterized by multi-markers.

## Highlights

• Six cold-pressed oils were stored for six months under household conditions.
• GC-FID, LC-MS/MS, and GC–MS tracked deterioration fingerprints over time.
• Polyphenols degraded rapidly, with p-coumaric acid dropping by 87% in 14 days.
• LASSO, RFR and correlation analysis identified oil-type-specific biomarkers.
• First systematic, multi-marker degradation analysis for oil stability prediction.

## Graphical abstract

**Figure:**
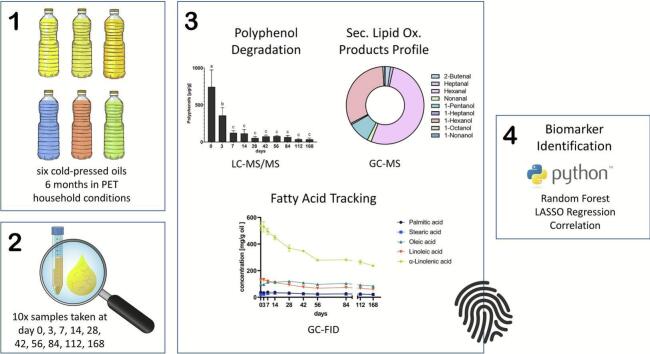

## 1 Introduction

Vegetable oils are increasingly recognized for their nutritional value due to their rich content of mono- and polyunsaturated fatty acids (MUFAs and PUFAs) (Koch et al., 2023). Their dietary consumption has been associated with various health benefits. Particularly, oils rich in PUFAs have been shown to positively influence blood pressure regulation and reduce the risk of coronary heart disease (Haque et al., 2024). However, adverse health effects such as hepatic inflammation may arise from the consumption of lipid oxidation products formed during processing or storage. Preventing oxidative degradation is therefore essential to ensure oil quality and safety (Böhm et al., 2013; Kanner, 2007).

Edible oils deteriorate via the two primary oxidative pathways, photooxidation and autoxidation due to oxygen exposure during storage (Pignitter et al., 2014; Tawfik & Huyghebaert, 1999). Both processes preferentially target unsaturated fatty acids, leading to the formation of volatile degradation products associated with rancidity. Under household conditions, light exposure represents a particularly important yet often underestimated factor. To mitigate oxidative deterioration, oils are typically stored under reduced light and oxygen exposure and at lower temperatures, while the presence of endogenous antioxidants such as tocopherols and polyphenols can further suppress oxidation (Choe & Min, 2006; Mehany, González-Sáiz, & Pizarro, 2025). Cold-pressed oils, although valued for their minimal processing and higher content of bioactive compounds, often contain elevated levels of PUFAs, rendering them especially susceptible to oxidative degradation (Gharby et al., 2025). Secondary lipid oxidation products such as aldehydes and ketones are largely responsible for the characteristic off-flavors and off-odors and therefore critically determine sensory quality and shelf-life (Geng, Liu, & Zhang, 2023; Grajzer et al., 2020).

Despite advances in analytical techniques predicting the oxidative stability and shelf-life of oils remains challenging due to the complex interplay of chemical, physical, and environmental factors (Karami, Rasekh, & Mirzaee – Ghaleh, 2020). A number of storage studies have been conducted to assess oil deterioration, primarily by tracking the formation of primary and secondary oxidation products under accelerated conditions (Cui et al., 2024; Holler et al., 2023; Pignitter et al., 2014; Tanouti et al., 2012). Among the most widely studied markers of oil aging are aldehydes such as hexanal, pentanal, and nonanal, which are derived from the oxidation of unsaturated fatty acids and serve as established indicators of rancidity (Parisi, 2016). Hexanal, in particular, has been proposed as a universal marker due to its sensitivity and prevalence in PUFA oxidation (Jacobsen, Let, Nielsen, & Meyer, 2008). However, its reliability as a sole marker has been questioned, as sensory rejection and hexanal levels do not always correlate well, especially across diverse oil matrices. For instance, Ampofo, Grilo, Langstaff, and Wang (2022) showed that walnut oil had strong correlation between hexanal and rancidity at low temperatures (r ≈ 0.92), but at ambient storage (23 °C), rancidity perception was associated with multiple other volatiles, reducing hexanal's predictive power. Tocopherols have also been investigated as early depletion markers, with their degradation kinetics suggested as predictive indicators of shelf-life (Bayram, Parra-Escudero, Decker, & Lu, 2024). In contrast, other antioxidant compounds such as polyphenols have rarely been studied in detail as biomarkers of oil stability, despite their established role in modulating lipid oxidation. This gap underscores the need for oil-specific degradation profiling that considers not only individual markers, but also the interplay of antioxidants and oxidation products over time.

While many studies focused on either fatty acid degradation, polyphenol depletion, or volatile accumulation in isolation, only few have integrated these chemical classes across extended storage to capture full deterioration pathways. This integration is critical to identify robust biomarkers that reflect both oxidative susceptibility and resistance. To address this, the present study employed a comprehensive analytical approach combining GC-FID for fatty acid profiling, LC-MS/MS for polyphenol quantification and GC–MS for monitoring secondary lipid oxidation products. The aim was to identify robust and oil-type-specific markers for oxidative deterioration in six commonly consumed cold-pressed oils under household conditions over a period of six months. To our knowledge, no previous study has combined multivariate machine learning (ML) approaches with multi-class chemical profiling to derive oil-type-specific deterioration biomarkers. To systematically assess the predictive value of each compound, we applied Least Absolute Shrinkage and Selection Operator (LASSO) and Random Forest regression (RFR). These approaches are widely recognized for their ability to perform feature selection in high-dimensional omics and food quality datasets (Li, Bai, Wang, & Ying, 2024; W. Zhang, Huang, Wang, Lehmann, & Chen, 2025). By integrating these tools with analytical data, we aim to deepen our understanding of lipid oxidation pathways and support the development of future detection strategies. While lipid oxidation is often assessed using individual markers such as peroxide value or hexanal concentration, such single-parameter approaches capture only isolated stages of the oxidative process. Given that oxidative deterioration proceeds from PUFA-driven initiation through antioxidant depletion to volatile formation, a multi-marker strategy integrating different chemical classes is required to describe oil stability under realistic storage conditions. Accordingly, this study does not aim to validate a single universal oxidation marker, but to establish oil-type-specific deterioration fingerprints by integrating fatty acids, polyphenols and secondary oxidation products.

## 2 Materials and methods

### 2.1 Chemicals and standards

Analytical-grade solvents and reagents were used throughout the study. Methanol, *n*-hexane, acetonitrile and methyl tert-butyl ether (MTBE) were of LC–MS or GC grade. They were obtained from established suppliers including Carl Roth GmbH (Karlsruhe, Germany), Sigma-Aldrich (Vienna, Austria) and VWR International GmbH (Vienna, Austria).

Reference standards (purity ≥95%) used for quantification were as follows: For fatty acid analysis (GC-FID): Methyl palmitate (C16:0), methyl stearate (C18:0), methyl oleate (C18:1), methyl linoleate (C18:2), methyl α-linolenate (C18:3), with methyl heptadecanoate (C17:0) as internal standard, for polyphenol profiling (LC-MS/MS): Kaempferol, quercetin-3-O-glucoside, naringenin, apigenin, epicatechin, secoisolariciresinol, caffeic acid, ferulic acid, p-coumaric acid, gallic acid, vanillic acid, 4-hydroxybenzoic acid, resveratrol and for secondary lipid oxidation products (SPME-GC–MS): Hexanal, 2-pentanone, 2-heptanone and 1-hexanol. Calibration parameters are shown in table S1–3

### 2.2 Oil samples and conditions of storage study

Six cold-pressed vegetable oils were selected based on their diversity, fatty acid composition and commercial relevance: black cumin (BC), canola (CA), sunflower (SF), high-oleic sunflower (HOSF), linseed (LS) and hempseed oil (HS). Each oil type was sourced from three independent Austrian producers to account for batch variability (Schalk Mühle, Weber Michl, and Hagenthaler Ölmanufaktur). A total of 18 oils, with three replicates each, were included in the storage study. For each oil type and time point, reported values reflect three independent commercial batches (suppliers) × three bottle replicates, resulting in *n* = 9 observations per time point. Fig. S1 shows the oil samples, which were stored in transparent polyethylene terephthalate (PET) bottles under controlled conditions (22 ± 2 °C, 12 h light exposure per day with cold fluorescent lighting (∼1000 lx)) to simulate typical household conditions. Samples were taken at ten predefined time points over six months (T0-T9 at day 0, 3, 7, 14, 28, 42, 56, 84, 112 and 168). At each time point, bottles were opened once, aliquots were withdrawn and stored at −80 °C until analysis.

### 2.3 Fatty acid analysis by GC-FID

The fatty acid composition of oils was determined as fatty acid methyl esters (FAMEs) using gas chromatography with flame ionization detection (GC-FID). Sample preparation followed the protocol from Lall, Proctor, and Jain (2009) with modifications by Pignitter et al. (2014) and Grüneis et al. (2019). Briefly, 100 mg oil was weighed into a 50 mL tube and spiked with 500 μL of a 1% heptadecanoic acid methyl ester (HME) internal standard. Derivatization was carried out with 2 mL toluene, 100 mg pyrogallol, and 4 mL sodium methoxide (0.5 M in methanol), incubated at 50 °C for 10 min under argon, and neutralized with 200 μL glacial acetic acid. Extraction was performed with 5 mL n-hexane and 5 mL H₂O, and the organic phase was dried over anhydrous Na₂SO₄ and filtered (0.45 μm PVDF).

Analyses were performed on a Shimadzu GC-2010 Plus system equipped with an AOC-20i autosampler and a ZB-WAX column (30 m × 0.25 mm × 0.25 μm). Injection volume was 1 μL (split 1:20) at 200 °C, with helium as carrier gas (3 mL/min). The oven program was 60 °C (2 min), ramp 13 °C/min to 150 °C, then 2 °C/min to 240 °C. The FID was operated with N₂ (30 mL/min), H₂ (40 mL/min) and air (400 mL/min). Samples were run in triplicate. Methyl palmitate, methyl stearate, methyl oleate, methyl linoleate and methyl linolenate were used for fatty acid quantification. Calibration parameters are shown in Table S1. Limits of detection (LOD) and quantification (LOQ) were calculated from calibration curve standard deviations.

### 2.4 Polyphenol profiling by LC-MS/MS

Polyphenols were extracted according to a modified protocol of Singleton and Rossi (1965). In brief, 3.5 g oil was extracted with 3.5 mL *n*-hexane and 1 mL methanol:H_2_O (80,20, *v*/v), vortexed (4 min), and centrifuged (6 min, 4 °C, 2280 g). The methanol phase was collected and pooled over three sequential extractions. After *n*-hexane washing, the combined methanol fractions were dried under nitrogen, freeze-dried, and reconstituted in 500 μL methanol (80%).

Polyphenols were analyzed based on Pointner et al. (2024) on a Shimadzu LCMS-8040 triple quadrupole system using a Kinetex C18 column (150 × 2.1 mm, 2.6 μm, Phenomenex, Aschaffenburg, Germany). Mobile phases were H_2_O with 0.1% formic acid (A) and acetonitrile with 0.1% formic acid (B). The gradient elution was: 10% B (0–2 min), 10–80% B (2–8 min), 80–100% B (8–20 min), followed by re-equilibration. Flow rate was 0.3 mL/min, injection volume 5 μL. Electrospray ionization was used in positive and negative mode at ±4.5 kV depending on fragmentation with a collision energy of ±30 V. N_2_ was used as nebulizing gas (3 L/min) and drying gas (10 L/min). The desolvation line and heat block temperatures were set to 150 °C and 350 °C, respectively. Ar was used as collision gas at 230 kPa. Multiple Reaction Monitoring (MRM) was applied in LabSolutions (v5.99 SP2, Shimadzu). A list of analyzed polyphenolic substances and *m/z* values is shown in Table S4. For polyphenol quantification, reference standards were used, including kaempferol, quercetin-3-O-glucoside, naringenin, apigenin, epicatechin, secoisolariciresinol, caffeic acid, ferulic acid, p-coumaric acid, gallic acid, vanillic acid, 4-hydroxybenzoic acid and resveratrol. These standards represent the major polyphenol classes (flavan-3-ols, flavones, flavonoids, lignans, phenolic acids, phenolic aldehydes and stilbenes) present in the investigated oils. Standard curve parameters are shown in Table S2. Signal-to-noise ratio thresholds were used for LOD (S/N ≥ 3) and LOQ (S/*N* ≥ 10) determination. Data evaluation and processing was done in Skyline (v23.0) (Pino et al., 2020).

### 2.5 Secondary lipid oxidation product analysis by SPME-GC–MS

Volatiles were determined via solid-phase microextraction (SPME) coupled with GC–MS based on Guillén, Cabo, Ibargoitia, and Ruiz (2005) and Holler et al. (2023). 2.5 g oil were weighed into sealed headspace vials, internal standards were added prior to analysis to account for matrix effects, and extracted using a DVB/CAR/PDMS fiber. Volatile compounds were analyzed using a Shimadzu GCMS-QP2010 Ultra equipped with a ZB-WAX column (30 m × 0.25 mm × 0.25 μm). Oven conditions were 40 °C (5 min), ramped at 4 °C/min to 280 °C. The injector was set to 220 °C in splitless mode (10 min fiber desorption). Mass spectra were recorded over *m/z* 40–400 at 70 eV. Compounds were identified using the NIST library (score > 0.85), and key volatiles such as hexanal, 1-hexanol, 2-heptanone and 2-pentanone were quantified by calibration curves, which are shown in Table S3. GC–MS data were further processed using OpenChrom software (v1.5.0).

### 2.6 Biomarker identification and statistical analysis

To identify robust biomarkers indicative of oil deterioration during storage, systematic multivariate statistical and ML approaches were applied to the data obtained from fatty acids, polyphenols and secondary lipid oxidation products. Biomarkers were defined as substances where concentrations changed significantly and consistently with storage time. All scripts and analysis pipelines used for biomarker identification are available via GitHub (https://github.com/TobiPointi/Deterioration-Biomarker-Analysis-in-Cold-Pressed-Oils.git). This approach ensures that selected biomarkers reflect robust, time-dependent deterioration trends rather than method-specific or oil-unspecific effects.

#### 2.6.1 Data normalization and preprocessing

For fatty acid data, centered log-ratio (CLR) transformation was applied to account for compositional constraints (Gloor, Macklaim, Pawlowsky-Glahn, & Egozcue, 2017). All datasets were normalized using z-score scaling (mean-centered and divided by standard deviation). Missing values, which mainly arose from concentrations below LOQ at early or late storage stages, were imputed using forward- and backward-filling strategies. Principal Component Analysis (PCA) was used to visualize sample clustering and variance explanation.

#### 2.6.2 Multivariate analysis and feature selection

Three core statistical models were used for feature selection. Least Absolute Shrinkage and Selection Operator (LASSO) Regression was used to penalize less informative variables (Ranstam & Cook, 2018). The regularization parameter α was dynamically adapted (typically 0.01), and a significance threshold of 20% of the maximum absolute coefficient was applied. Random Forest Regression (RFR) is an ensemble ML method that was applied to rank features by their importance in predicting storage time (Breiman, 2001; Ho, 1995). A total of 100 estimators were used, and a dynamic significance threshold of 10% of the maximum importance score was applied. RFR is used as a key tool in feature ranking and biomarker discovery in foodomics and other high-dimensional datasets (Li et al., 2024). Spearman correlation as a non-parametric test between compound concentrations and storage time was calculated to assess monotonic trends. Each compound was evaluated across all three models per oil type and compound class (fatty acids, polyphenols, volatiles). Identified biomarkers reflect statistically robust associations with storage time and oxidative progression and do not imply direct mechanistic causality for all compounds.

#### 2.6.3 Composite scoring and ranking

A balanced weighting was applied to reflect both model robustness and complementarity. RFR and LASSO were prioritized (40% each) as established feature selection approaches in high-dimensional biomarker discovery, together with correlation (20%) due to its univariate nature and greater susceptibility to spurious associations. Each metric was normalized to a 0–1 scale using min–max normalization, and the final biomarker score was calculated for every substance across all oil types. The highest-scoring compounds were considered the most reliable indicators of oxidative deterioration.

### 2.7 Statistical testing

All experiments were performed with at least three independent analytical replicates. Outliers were screened using Grubbs' test. Data are presented as mean ± standard deviation. Temporal trends during storage were evaluated using descriptive statistics and correlation analysis. Statistical tests were applied in GraphPad Prism 10.4.1. One-way ANOVA followed by Tukey's post-hoc test was used to assess significance of concentration changes across time points. For non-parametric comparisons, the Mann–Whitney *U* test was applied. Results were considered statistically significant at *p*-values <0.05.

## 3 Results

### 3.1 Fatty acid composition by GC-FID

To provide a baseline for subsequent degradation biomarker analysis, the fatty acid composition of the six analyzed cold-pressed oils was determined over 168 days of storage. This characterization is essential, as oxidative susceptibility of edible oils is primarily governed by the proportion of PUFAs, while MUFAs and SFAs contribute to oxidative stability and compositional context (Gharby et al., 2025). Accordingly, linoleic and α-linolenic acids were considered the principal fatty-acid-derived drivers of oxidation in subsequent analyses, whereas oleic acid and SFAs were evaluated as stabilizing matrix components.

#### 3.1.1 Initial fatty acid distribution

The initial fatty acid composition (day 0) of the six cold-pressed oils black cumin (BC), high-oleic sunflower (HOSF), canola (CA), sunflower (SF), linseed (LS), and hempseed (HS) is shown in Fig. S2. The profiles varied substantially among the oils.

BC was characterized by a high linoleic acid content (67.87 ± 4.27%), together with palmitic acid (14.31 ± 0.99%). CA contained high oleic acid (55.40 ± 2.31%), linoleic acid (25.84 ± 1.03%), and α-linolenic acid (11.28 ± 0.51%). SF was dominated by linoleic acid (63.56 ± 2.35%). HOSF showed very high oleic acid levels (83.10 ± 3.87%), as expected for this type of seed. LS exhibited the highest α-linolenic acid content (65.37 ± 4.02%), while HS presented a balanced polyunsaturated profile with linoleic acid (60.02 ± 2.60%) and α-linolenic acid (22.00 ± 1.16%).

#### 3.1.2 Changes in fatty acids during storage

**Fig. 1:**
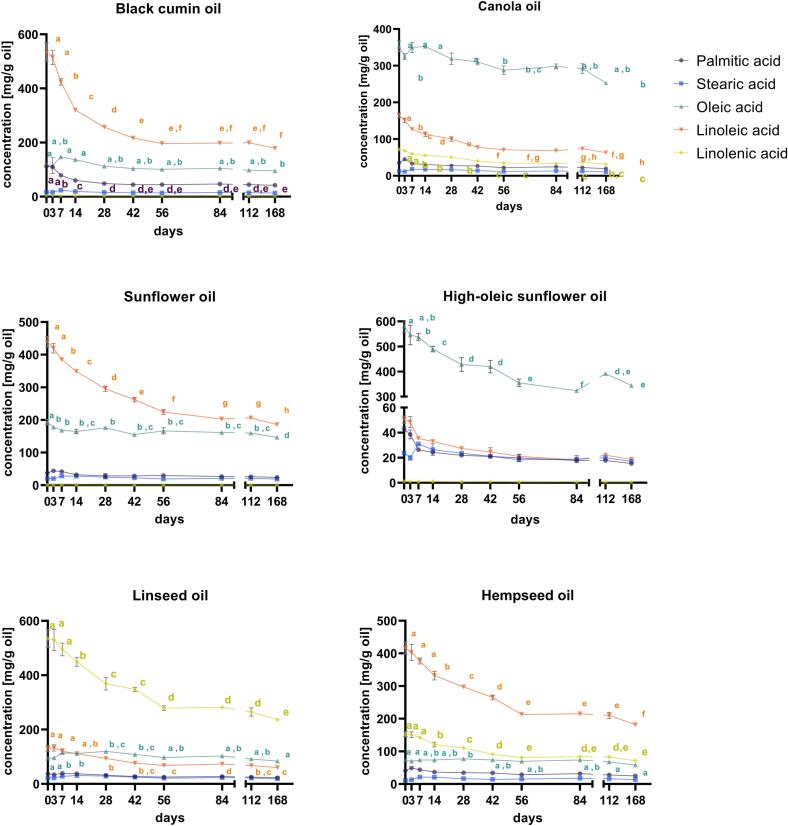
Changes in fatty acid composition [mg/g oil] during 168 days of storage for the six cold-pressed oils under household conditions: **(A)** black cumin, **(B)** canola, **(C)** sunflower, **(D)** high-oleic sunflower, **(E)** linseed and **(F)** hempseed oil. One*-*way ANOVA with Tukey's post hoc test was conducted. Statistical differences are indicated with different lowercase letters (p < 0.05). Each data point represents mean values and error bars represent SD (n = 9) and are in some cases smaller than symbol size.

Significant changes in fatty acid composition occurred over the 168-day storage period under simulated household conditions, primarily reflecting oxidative degradation of unsaturated fatty acids. The magnitude and kinetics of these changes differed by oil type. The fatty acid degradation data is shown in Fig. 1.

In BC, linoleic acid decreased by 66.5% over the storage period, while saturated fatty acids (SFAs) remained more stable. CA showed decreases of 61.5% in linoleic acid, 55.9% in α-linolenic acid, and 28.2% in oleic acid. SF exhibited a 57.7% reduction in linoleic acid, while HOSF displayed a 40.8% decline in oleic acid, with smaller changes in PUFAs. LS, initially rich in α-linolenic acid, showed a 55.8% decline and a 53.6% reduction in linoleic acid, while MUFAs and SFAs remained relatively stable. HS showed decreases in both linoleic and α-linolenic acid, whereas oleic acid and SFAs remained constant. Overall, oils with higher PUFA content (BC, LS, HS) exhibited the steepest reductions in fatty acids, while oils with lower PUFA proportions (CA, SF, HOSF) showed more moderate declines. Across all oils, the largest absolute and relative decreases occurred in PUFAs, consistent with their higher reactivity and role as primary oxidation substrates under light exposure. In contrast, SFAs changed only modestly and are therefore interpreted as contextual indicators rather than mechanistic drivers of oxidation. The full list of the fatty acids analyzed at the ten time points is provided in Table S5.

### 3.2 Polyphenol analysis by LC-MS/MS

Polyphenols in edible oils serve as important antioxidants that inhibit and delay lipid oxidation by scavenging free radicals and chelating pro-oxidative metals, thus playing a key role in shelf-life stability. Their profiles and concentrations were analyzed via LC-MS/MS in MRM mode.

#### 3.2.1 Initial Total polyphenol content and class distribution

At the beginning of storage, the six cold-pressed oils showed clear differences in total polyphenol content as shown in Fig. S3. BC contained the highest levels with 745.1 ± 227.2 μg/g, significantly exceeding all other oils (*p* < 0.05). The other oils (CA, SF, HOSF, LS, HS) showed lower concentrations ranging from 68.9 to 150.7 μg/g.

Polyphenols were grouped into phenolic acids, flavonoids, flavones, flavan-3-ols, lignans, and stilbenes. The relative distribution of these classes is shown in Fig. S4. BC exhibited the most complex composition, dominated by phenolic acids (80%), with smaller contributions from flavonoids (12.5%), flavones (5.5%), and small amounts of flavan-3-ols and lignans. Stilbenes and phenolic aldehydes were only detected in BC. CA also contained predominantly phenolic acids (53%) and a notable fraction of flavones (28.1%). SF showed the highest proportion of flavones (51.6%). LS contained high flavone levels (44.3%) along with some lignans (7%). HS displayed a more diverse profile, with phenolic acids (43.8%), flavonoids (24.4%), flavones (18.6%), and lignans (9%).

#### 3.2.2 Polyphenol degradation over storage time

**Fig. 2:**
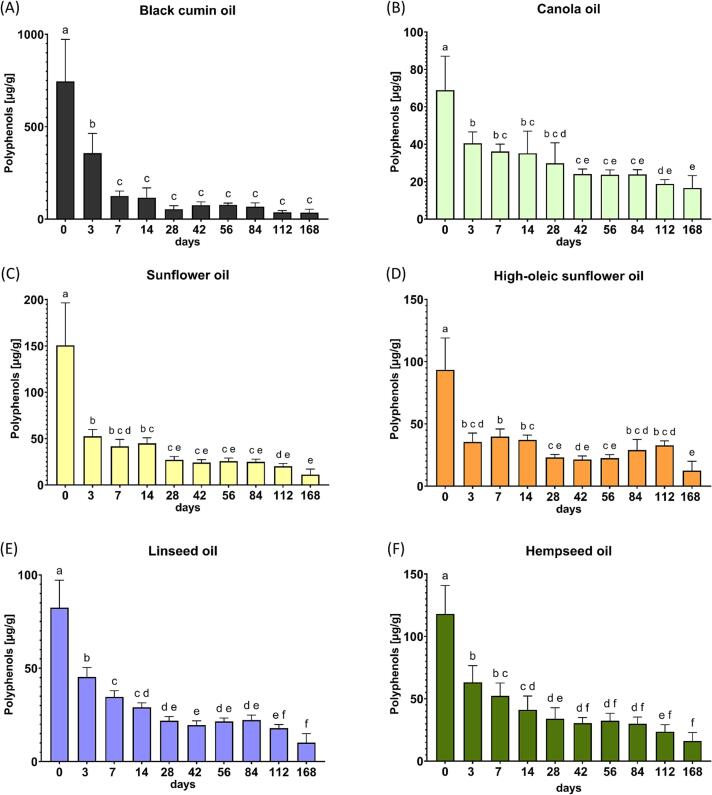
Change of total polyphenol content [μg/g oil] in six cold-pressed oils during 168 days of storage, measured via LC-MS/MS*.***(A)** black cumin, **(B)** canola, **(C)** sunflower, **(D)** high-oleic sunflower, **(E)** linseed and **(F)** hempseed oil. *O*ne-way ANOVA with Tukey's post hoc test was conducted. Statistical differences are indicated with different lowercase letters (p < 0.05). Each data point represents the mean *+ SD, n ≥ 9.*

The antioxidant activity of polyphenols leads to their gradual decline during storage, as these compounds neutralize lipid-derived radicals and chelate metal ions (Shahidi & Ambigaipalan, 2015). All six cold-pressed oils showed a significant decrease in total polyphenol content across the 168-day storage period, although the extent and rate of decline varied by oil type, which is shown in Fig. 2. BC exhibited the steepest decline, with a marked reduction already evident at day 3. By day 168, 95% of the initial polyphenols were degraded, resulting in 34.8 ± 17.5 μg/g oil at the end of storage. Despite this loss, BC maintained the highest residual polyphenol concentration throughout the study. CA showed a gradual, near-linear decrease over time. HOSF, with lower initial phenolic levels, exhibited an early reduction within the first 3 days. SF, LS, and HS followed similar patterns, with significant decreases detected from day 3 onward. By day 84, all three had lost more than 50% of their initial polyphenol content.

**Fig. 3:**
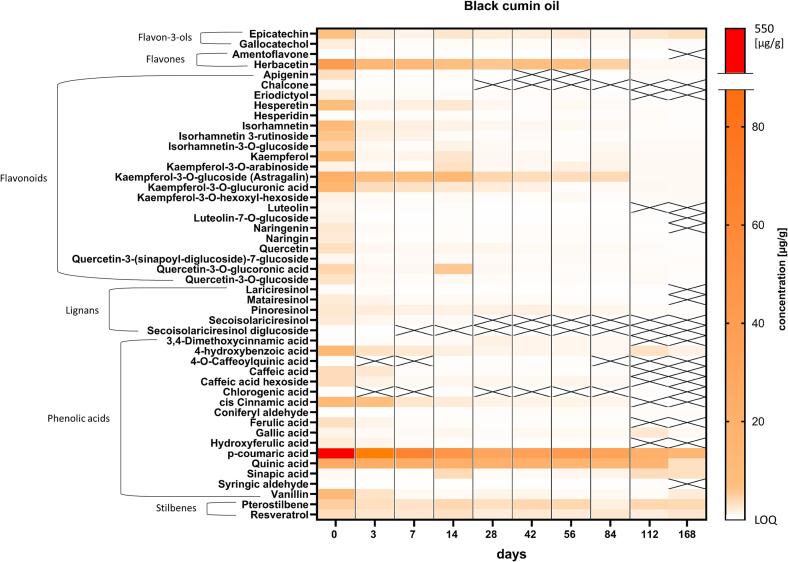
Heatmap of quantified polyphenols in black cumin oil across six months storage. The color scale indicates different concentrations [μg/g oil] and the different specific polyphenolic substances identified and quantified were put into polyphenolic classes. Crossed-out cells are ≤ LOQ. Values are shown as mean, *n ≥ 9.*

In most oils, phenolic acids exhibited the earliest and steepest decreases, significant from day 3 onward, whereas flavonols, flavones, and lignans showed comparatively slower declines. Fig. 3 provides a heatmap of all 48 quantified polyphenols in BC, grouped by class, across storage. Phenolic acids were initially dominant and exhibited the steepest declines during storage. For instance, p-coumaric acid decreased from 538.2 to 13.2 μg/g (−97.5%), and quinic acid from 25.3 to 2.42 μg/g (−90%). The visualization highlights the rapid depletion of phenolic acids within the first weeks, while flavonoids, lignans, and stilbenes persisted longer.

The full dataset of all quantified polyphenolic compounds is provided in Table S6. In CA, sinapic acid exhibited an early transient increase before declining to low residual levels by day 168 (−80%). Ferulic acid and ellagic acid also decreased strongly. Herbacetin, as the most abundant flavone, decreased from 19.1 to 1.41 μg/g (−93%). In SF, ellagic acid dropped from 16.7 to 0.45 μg/g (−97%), while quercetin-3-O-glucoside decreased from 1.39 to 0.13 μg/g (−91%) and in HOSF, ellagic acid decreased from 10.4 to 0.47 μg/g (−95%). Caffeic acid and its hexoside showed rapid early-phase depletion. In LS, hydroxycinnamates showed rapid loss, with p-coumaric acid decreasing from 6.07 μg/g to 0.53 μg/g by day 168 (−92%). Several linseed specific lignans such as secoisolariciresinol also diminished by about 80% over the six months study period. In HS, p-coumaric acid decreased from 7.2 to 0.39 μg/g (−95%), while kaempferol conjugates and lignans showed more gradual mid-term declines.

Overall, large quantitative differences and specific polyphenolic fingerprints were observed across oils, with BC showing the highest initial levels but also the steepest absolute reductions, while CA, SF, HOSF, LS and HS exhibited distinct compound-specific degradation patterns. Together, these findings demonstrate that polyphenolic degradation is not only concentration-dependent but also matrix-specific. Phenolic acids generally showed the fastest decay, while flavonoids and lignans tended to degrade more slowly. These insights support the strategic use of specific polyphenols as early or mid-term markers of oil stability.

### 3.3 Secondary lipid oxidation products via SPME-GC–MS

**Fig. 4:**
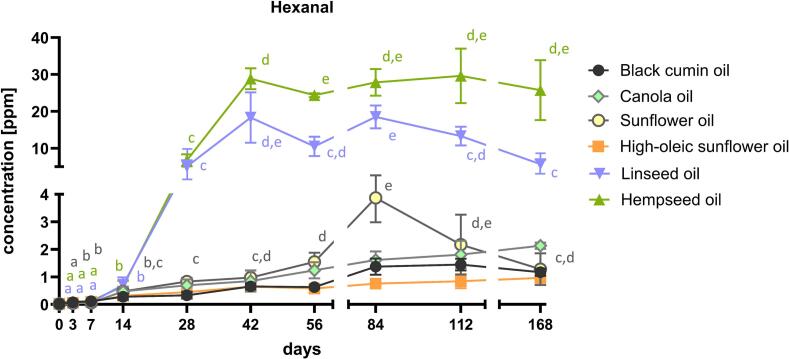
Hexanal concentration in ppm over 168 days of storage in the six cold-pressed oils analyzed. One-way ANOVA with Tukey's post hoc test was conducted. Statistical differences are indicated with different lowercase letters (p < 0.05). The data points represent values of mean ± SD, n ≥ 9.

Volatile compounds formed by secondary lipid oxidation are key indicators of oil deterioration and were monitored via SPME-GC–MS. Among the analyzed oils hexanal was quantified over 168 days as shown in Fig. 4. The formation was oil-dependent. LS and HS showed the strongest increases. LS rose to 18.3 ppm after 42 days, while HS increased to 6.7 ppm after 28 days already, reached 28.9 ppm after 42 days, and remained >24 ppm thereafter. Both oils followed between day 14 and day 42 an exponential, pseudo-first-order pattern in the propagation phase:

(1) $$C_{\mathrm{LS}}\left(t\right)=0.08*e^{0.15*\left(t-14\right)},\left(R^{2}=0.998\right)$$

(2) $$C_{\mathrm{HS}}\left(t\right)=0.11*e^{0.14*\left(t-14\right)},\left(R^{2}=0.991\right)$$

where C(t) is the hexanal concentration [ppm] at time t [days].

By contrast, the other oils stayed an order of magnitude lower over the whole study. SF exhibited a transient maximum of 3.9 ppm after 84 days followed by a decrease to 1.28 ppm at day 168. BC rose modestly to 1.45 ppm by day 112, HOSF gradually to 0.96 ppm and CA to 2.13 ppm by the end of storage.

Across the different oil types tested distinct fingerprints for unsaturated alkenals (e.g., (E)-2-nonenal, (E)-2-decenal), conjugated dienals (2,4-heptadienal, 2,4-decadienal), saturated aldehydes (heptanal, nonanal), furans (2-pentyl-furan, 5-ethyl-2(5)-furanone), allylic alcohols (2-octen-1-ol, 2-decen-1-ol, 1-octen-3-ol), and advanced markers as e.g. 4,5-epoxy-(E)-2-decenal were evaluated. All detected and identified secondary lipid oxidation compounds over time of storage expressed either as quantified concentrations [ppm] or semi-quantified area under the curve [AuC] values are reported in Table S7 and S8. In total 20 secondary lipid oxidation products could be identified.

In BC, nonanal and the electrophilic aldehyde 4,5-epoxy-(E)-2-decenal increased most prominently, showing 10.3- and 13.9-fold rises, respectively, by the end of storage, while heptanal rose 6.5-fold. Unsaturated aldehydes such as (E)-2-decenal and (E)-2-octenal accumulated steadily, whereas 5-ethyl-2(5)-furanone showed a transient maximum around day 112 (3.1-fold increase). In CA, conjugated dienals were dominant, with 2,4-decadienal rising 13-fold by day 112. Moderate increases were also observed for (E)-2-nonenal, 2,4-heptadienal and heptanal. SF showed a similar aldehyde profile, with a 13.1-fold increase in 2,4-decadienal and 3.7-fold in (E)-2-nonenal peaking after 112 days, while other dienals rose earlier. In HOSF, nonanal was the most dynamic marker, increasing nearly 5-fold within 42 days. Decanal also rose sharply by day 84, while (E)-2-decenal increased mid-storage. LS was characterized by a rise of 2-pentyl-furan, which peaked at day 112 with 28.9-fold higher levels and remained elevated thereafter. Conjugated dienals such as 2,4-heptadienal and 2,4-decadienal also accumulated, together with nonanoic acid, while 1-octen-3-ol declined rapidly. In HS, (E)-2-heptenal showed the most increase by 42.7-fold by day 84. Substantial increases were also seen for 2-pentyl-furan, 2,4-decadienal, and nonanoic acid, alongside moderate rises in (E)-2-nonenal, heptanal and 5-ethyl-2(5)-furanone. These measurements highlight distinct, oil-specific volatile fingerprints that underpin the biomarker selection discussed in the next section.

### 3.4 Biomarker identification from oil-specific fingerprints

To identify reliable indicators of oil deterioration, a comprehensive biomarker analysis was conducted using combined datasets from fatty acids (GC-FID), polyphenols (LC-MS/MS), and secondary lipid oxidation products (GC–MS). Each dataset was subjected to LASSO, RFR, and Spearman correlation. These were normalized (z-score), weighted and aggregated to generate a final ranking of potential biomarkers per oil type. This integrative strategy revealed distinct fingerprints of deterioration for each oil, comprising substances with the most time-responsive behavior across chemical classes. PCAs were performed on each dataset of fatty acids, polyphenols and volatiles to assess clustering patterns and variance explained. Fig. S5 illustrates the PCA scores and loadings for fatty acids in BC, showing a clear separation of storage stages along PC1 and PC2, supporting the robustness of the biomarker selection approach. The top biomarkers per oil type are listed in Table 1.

**Table 1:** Results of the top biomarker identification for each oil type with weighted score.

Oil Type	Substance Type	Substance	Weighted Score	Trend over Time
Black cumin oil	Fatty Acid	Linoleic acid	0.68	↓
	Fatty Acid	Stearic acid	0.65	↓
	Volatile	2-Decen-1-ol	0.55	↑
	Volatile	(E)-2-Nonenal	0.54	↑
	Volatile	2-Octen-1-ol	0.42	↑
	Polyphenol	Matairesinol	0.28	↓

Canola oil	Volatile	2,4-Decadienal	0.95	**↑**
	Fatty Acid	Oleic acid	0.89	**↓**
	Fatty Acid	Linoleic acid	0.58	**↓**
	Fatty Acid	α-Linolenic acid	0.52	**↓**
	Volatile	2,4-Heptadienal	0.50	**↑**
	Polyphenol	Sinapic acid	0.37	**↓**
	Volatile	2,4-Decadienal	0.91	**↑**

Sunflower oil	Fatty Acid	Linoleic acid	0.86	**↓**
	Fatty Acid	Oleic acid	0.57	**↓**
	Volatile	2,4-Heptadienal	0.41	**↑**
	Polyphenol	Quercetin-3-O-glucoronic acid	0.32	**↓**

HO sunflower oil	Volatile	Decanal	0.96	**↑**
	Fatty Acid	Stearic acid	0.84	**↓**
	Fatty Acid	Linoleic acid	0.78	**↓**
	Polyphenol	Caffeic acid	0.50	**↓**
	Polyphenol	Quinic acid	0.33	**↓**

Linseed oil	Fatty Acid	α-Linolenic acid	0.78	**↓**
	Volatile	1-Hexanol	0.56	**↑**
	Fatty Acid	Oleic acid	0.44	**↓**
	Volatile	1-Octen-3-ol	0.43	**↑**
	Polyphenol	p-Coumaric acid	0.40	**↓**

Hempseed oil	Fatty Acid	Linoleic acid	1.000	**↓**
	Volatile	1-Hexanol	0.46	**↑**
	Volatile	2,4-Decadienal	0.42	**↑**
	Volatile	(E)-2-Heptenal	0.36	**↑**
	Polyphenol	Chlorogenic acid	0.33	**↓**

For BC, linoleic acid was the fatty acid-derived marker with the highest score, reflecting the fatty acid degradation, while 2-decen-1-ol, (E)-2-nonenal and 2-octen-1-ol stood out among volatile oxidation products. Additionally, matairesinol emerged as the most relevant polyphenol marker, confirming the susceptibility of lignans to degradation in this oil type. For CA, the lipid oxidation products 2,4-decadienal and 2,4-heptadienal were most prominent, followed by the fatty acids oleic acid, linoleic acid, and α-linolenic acid. Sinapic acid was highlighted as the key polyphenol biomarker, aligning with its well-known role in canola oil stability (Koski, Pekkarinen, Hopia, Whl, & Heinonen, 2003). In SF, the volatile aldehydes 2,4-decadienal and 2,4-heptadienal dominated the biomarker fingerprint, while linoleic acid and oleic acid were consistent fatty acid markers. Quercetin-3-O-glucuronic acid was identified as the most reliable polyphenolic marker. In HOSF, decanal dominated the biomarker profile, indicating its strong link to oxidative rancidity in this oil, while caffeic acid and quinic acid were identified as the top polyphenol-related markers. For LS, α-linolenic acid was the strongest biomarker, consistent with the high susceptibility of this highly unsaturated fatty acid to oxidation. Volatile alcohols such as 1-hexanol and 1-octen-3-ol were also highlighted, alongside p-coumaric acid as polyphenolic marker. Finally, HS was best described by the decline of linoleic acid, as the strongest biomarker across all oils (weighted score = 1.0). In addition, 1-hexanol, 2,4-decadienal and (E)-2-heptenal contributed to the volatile fingerprint, while chlorogenic acid was identified as the most significant polyphenol marker.

Beyond the top biomarkers found, the full weighted score tables for fatty acids, polyphenols, and secondary lipid oxidation products are in Tables S1–S3. Overall, fatty acids tended to receive higher weighted scores, reflecting their strong and consistent contribution to oil deterioration across storage conditions. In contrast, polyphenols generally showed lower scores, which can be explained by their high chemical diversity and oil-specific relevance rather than universal predictive power. Secondary lipid oxidation products occupied an intermediate position, with several aldehydes and alcohols emerging as robust markers in specific oils. These patterns underline that a combined fingerprint across fatty acids and volatiles provides the most reliable biomarker set, while polyphenols add important oil-specific resolution. Taken together, the fatty acid profiles define the intrinsic oxidative susceptibility of each oil, which subsequently governs antioxidant depletion kinetics and the formation of secondary oxidation products during storage.

## 4 Discussion

Oxidative deterioration of edible oils follows a temporally and mechanistically structured process. In the present study, this progression is captured across three complementary chemical layers. The fatty acid composition defines the intrinsic susceptibility to oxidation, polyphenols modulate the rate and trajectory of deterioration through antioxidant depletion and secondary lipid oxidation products accumulate as terminal indicators of rancidity. By integrating GC-FID, LC-MS/MS, and GC–MS data with ML-based ranking approaches (LASSO, RFR and Spearman), it was possible to identify oil-type-specific biomarker fingerprints that provide mechanistic insights into oxidative stability and deterioration. The following sections discuss lipid oxidation as a sequence of initiation, propagation, and termination.

### 4.1 Role of fatty acid composition in oxidative stability

Fatty acids consistently received the highest biomarker scores across nearly all oils, highlighting their central role as primary substrates for lipid oxidation. Consistent with established oxidation theory, oils with higher PUFA content exhibited greater oxidative susceptibility, whereas MUFA-rich oils showed increased resistance. SFAs, while not oxidation drivers, contributed to matrix stability and enabled comparative interpretation across oil types(Choe & Min, 2006; Koch et al., 2023). Although SFAs are chemically less susceptible to oxidation, they can still rank as statistical biomarkers when their concentrations show small but highly consistent temporal shifts or when they track compositional restructuring during prolonged storage, but mechanistically, PUFAs remain the primary oxidation substrates. Therefore, oils rich in α-linolenic acid (LS, HS) or linoleic acid (BC, SF) deteriorated fastest, reflected by steep PUFA losses and sharp rises in aldehydic volatiles (e.g., hexanal, 2-pentylfuran). Similar kinetics have been reported for flaxseed and other n-3–rich oils stored under light/air exposure (Cui et al., 2024; Grajzer et al., 2020; Tanouti et al., 2012). In contrast, high-oleic sunflower oil (HOSF) demonstrated markedly higher oxidative stability, consistent with its reduced PUFA fraction and enrichment in oleic acid. Previous studies have also shown that high-oleic cultivars maintain lower peroxide values and volatile aldehyde formation compared to conventional sunflower oil (Holler et al., 2023; Pignitter et al., 2014). Collectively, these results confirm that baseline susceptibility is largely encoded by the degree of unsaturation.

### 4.2 Polyphenol degradation

Previous olive oil studies reported 40–60% polyphenol losses over 12 months in darkness (Castillo-Luna, Criado-Navarro, Ledesma-Escobar, López-Bascón, & Priego-Capote, 2021). By contrast, under light exposure conditions, BC with the highest initial phenolic concentration (745 μg/g oil) exhibited rapid early loss of −85% of its major phenolic substance p-coumaric acid after just 14 days, underscoring the accelerating effect of light exposure and transparent packaging. This pattern mirrors prior observations that classically reactive phenolics act as sacrificial antioxidants during propagation/termination steps (Böhm et al., 2013; Kanner, 2007; Toma, Olah, Vlase, Mogoșan, & Mocan, 2015) and agrees with earlier findings in black cumin seed extracts, where phenolic acids were consumed rapidly during oxidative challenges (Ahmad et al., 2013).

Despite its steep decline, BC retained the highest residual phenolic levels after 6 months, supporting its relative stability compared to other PUFA-rich oils. Similar roles of specific phenolics were observed in other oils. Caffeic acid in HOSF and sinapic acid in CA were identified as relevant biomarkers, both of which have been reported as effective antioxidants in lipid matrices (Shahidi & Ambigaipalan, 2015). In canola oil, sinapic acid and its derivatives are well-documented contributors to oxidative stability, though they also undergo rapid photooxidative degradation in transparent packaging (Koski et al., 2003).

Taken together, our findings show that fatty acid composition sets the baseline susceptibility to oxidation, while polyphenols modulate the rate and trajectory of deterioration. Oils with both high PUFA content (e.g., HS, LS) and lower phenolic reserves were most vulnerable, whereas oils with either high MUFA (HOSF) or high phenolic content (BC) resisted deterioration more effectively.

### 4.3 Secondary lipid oxidation products as functional biomarkers

Secondary lipid oxidation products, particularly volatile aldehydes, emerged as strong and oil-type-specific markers. Compounds such as nonanal, 2,4-decadienal, and (E)-2-nonenal showed marked increases in several oils, consistent with their low odor thresholds and established role as indicators of rancidity (Crackel, Gray, Booren, Pearson, & Buckley, 1988). Hexanal, often used as a universal marker of PUFA oxidation (Jacobsen et al., 2008; Sharma et al., 2022), accumulated exponentially in LS and HS. The pseudo-first-order fit was valid during the propagation phase from day 14 to 42, reflecting rapid exponential growth in line with their high PUFA content, while later stages seemed to deviate due to substrate depletion and secondary reactions. These kinetics support previous reports that hexanal associates with sensory rejection in PUFA-rich oils and provide quantitative evidence that ∼1 ppm may serve as a practical threshold value for early detection of rancidity in household-stored oils (Clarke, O'Sullivan, Kerry, & Kilcawley, 2020; Gharby et al., 2025; Warner & Eskin, 1995). Nevertheless, as shown in olive and walnut oil studies (Ampofo et al., 2022), hexanal alone cannot fully explain rancidity perception across matrices.

### 4.4 Systematic biomarker identification

While PUFA-derived oxidation is the primary initiator of deterioration, reliance on a single chemical marker is insufficient to describe oil stability across diverse lipid matrices. Single-marker approaches, such as reliance on hexanal or peroxide value alone, capture only isolated stages of oxidation and often fail to generalize across oil matrices with differing fatty acid and antioxidant profiles. In contrast, the integrated multi-marker strategy applied here reflects the full oxidative trajectory and enables robust, oil-type-specific deterioration fingerprints that are mechanistically interpretable. By combining datasets from fatty acids, polyphenols, and volatiles, and applying ML–based feature selection, this study provides a new framework for biomarker discovery in edible oils. Similar multivariate and data-driven approaches have been successfully applied in metabolomics and foodomics for biomarker identification (Li et al., 2024; W. Zhang et al., 2025). The resulting hierarchy in our study showing that fatty acids dominating biomarker scores, followed by secondary volatiles, and polyphenols contributing lower but meaningful scores reflects the mechanistic sequence of deterioration. PUFA oxidation initiates instability, antioxidant depletion modulates the rate, and aldehyde accumulation serves as a terminal indicator of rancidity. The oil-type-specific fingerprints emphasize distinct vulnerabilities, such as rapid polyphenol depletion in black cumin oil, steep PUFA oxidation in linseed and hempseed oils, and aldehyde accumulation in sunflower and canola oils.

### 4.5 Study limitations and outlook

The novelty of this study lies in the systematic comparison of six cold-pressed oils from three different suppliers under household conditions to identify oil-type specific deterioration biomarkers. Our study design allowed us to capture the rapid degradation of light-sensitive antioxidants, with polyphenol losses already evident in the early days of storage. This effect was repeatedly demonstrated in other edible oils stored under illumination (Holler et al., 2023; Pignitter et al., 2014). By integrating chemical classes across methods and applying statistical learning, our study extends this work and demonstrates that deterioration cannot be reliably predicted by a single marker. Instead, robust oil-type-specific fingerprints emerge, offering a more nuanced and reliable approach to shelf-life assessment.

Limitations include the absence of other packaging materials (e.g., amber glass), which are known to provide greater light protection (Castillo-Luna et al., 2021; Grajzer et al., 2020). In addition, the study did not include direct sensory validation or accelerated storage tests, which would strengthen correlations between chemical markers and consumer perception. Future work should therefore validate the proposed biomarker fingerprints across different packaging materials and link them to sensory rejection thresholds in order to establish consumer-relevant cutoffs. This study focused on comparative oxidative behavior and biomarker identification across representative commercial cold-pressed oils rather than exhaustive population-level characterization. Variability related to cultivar, geographic origin and processing conditions was therefore not controlled but intentionally incorporated through the use of independent suppliers. While this approach enhances real-world relevance, future studies could further refine biomarker performance by including controlled varietal.

## 5 Conclusion

The present study provides an integrated comprehensive analysis of lipid oxidation in six cold-pressed oils, combining big data sets of GC-FID, LC-MS/MS and GC–MS measurements to identify oil-type-specific deterioration biomarkers. Fatty acid composition primarily determines oxidative susceptibility, while phenolic antioxidants modulate the rate and secondary volatiles mark the endpoint of degradation. The resulting fingerprints over half a year of storage capture the mechanistic sequence of deterioration and explain oil-specific oxidative stability differences under realistic household storage conditions.

In this study determined oil-type-specific deterioration biomarkers are not only beneficial for further knowledge of lipid oxidation pathways but also lay the groundwork for predictive shelf-life models. Integration of the proposed biomarkers with rapid, non-invasive screening technologies such as near-infrared spectroscopy (NIR) or electronic noses (e-nose), combined with ML modeling could offer promising applications for real-time quality, monitoring in industrial and domestic contexts. Such methods could significantly contribute to reducing food waste by enabling early detection of oxidative spoilage.

## Declaration of generative AI and AI-assisted technologies in the manuscript preparation process

During the preparation of this work, the authors used ChatGPT (GPT-5, OpenAI, San Francisco, USA) to improve the structure and language of the manuscript. It was also used to assist with debugging and code error handling. DeepL.com (v. 3.5, DeepL SE, Cologne, Germany) was used for language refinement and translation of sections originally drafted in German. After using these tools, the authors carefully reviewed and edited all content to ensure accuracy and take full responsibility for the integrity and originality of the published work.

## CRediT authorship contribution statement

**Tobias Pointner:** Writing – original draft, Visualization, Methodology, Investigation, Formal analysis, Data curation. **Philipp Steinlechner:** Visualization, Methodology, Investigation, Formal analysis, Data curation. **Marc Pignitter:** Writing – review & editing, Visualization, Supervision, Resources, Project administration, Funding acquisition, Conceptualization.

## Declaration of competing interest

The authors declare that they have no known competing financial interests or personal relationships that could have appeared to influence the work reported in this paper.

## Data Availability

Data will be made available on request.

## Appendix A Supplementary data

Supplementary material Image 1
