# Supplementary material for: Fatty acids, polyphenols and volatiles as predictive biomarkers of cold-pressed oil stability
Source: Food Chem X. 2026 Feb 13;34:103663. doi: 10.1016/j.fochx.2026.103663 (PMC12924181; doi:10.1016/j.fochx.2026.103663)
Supplement: Supplementary file 1 — Supplementary material [file mmc1.docx]

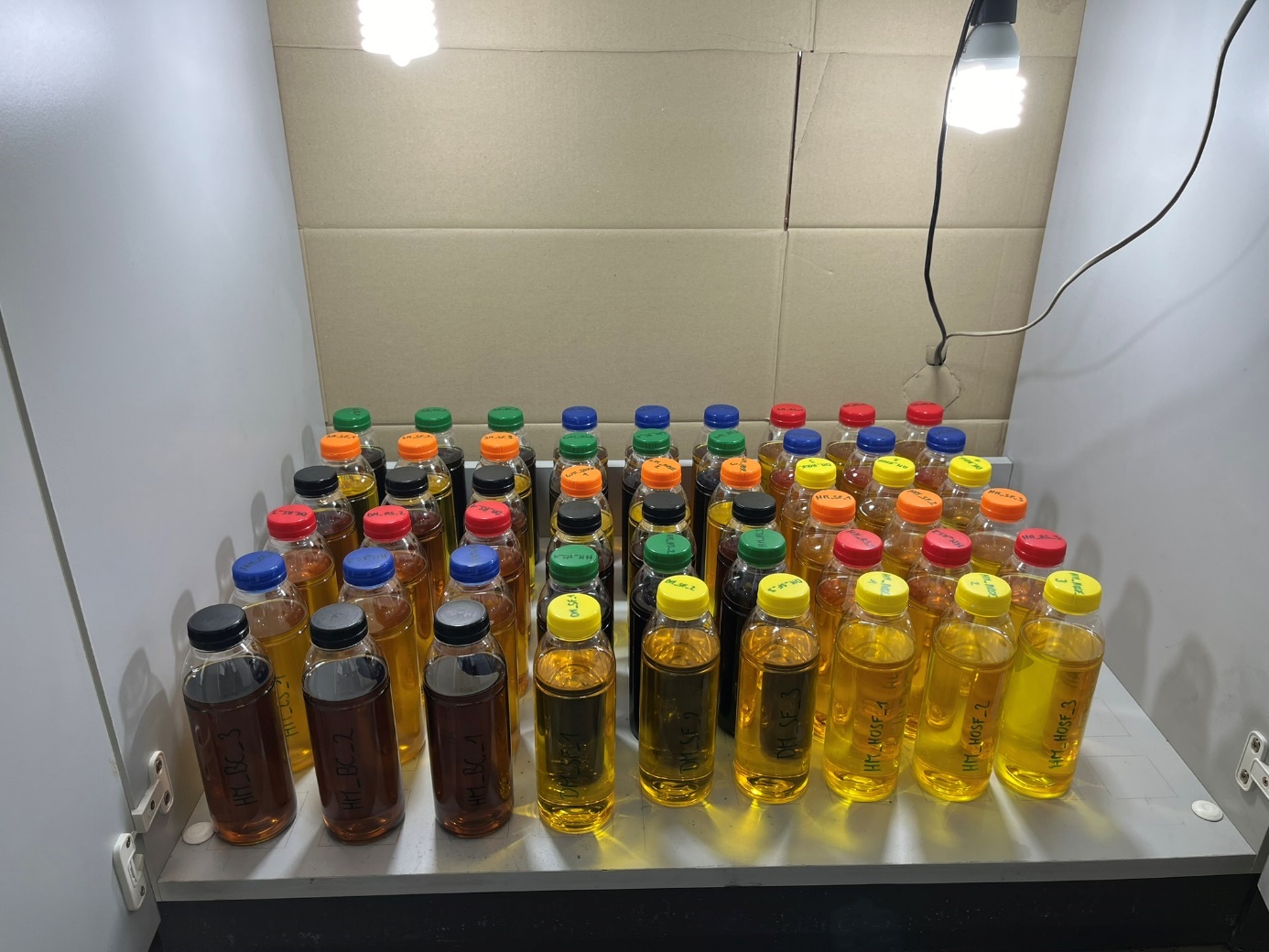


**Fig. S1**: *Setup of storage study with 54 oil samples (black cumin, canola, sunflower, high-oleic sunflower, linseed and hempseed oil from three different suppliers in triplicates) in PET bottles stored at room temperature for 12 h under cold fluorescent light per day for six months.*


**Fig S2:** *Initial relative fatty acid composition [%] (at day 0) of each oil:* *(A) black cumin, (B) canola, (C) sunflower, (D) high-oleic sunflower, (E) linseed and (F) hempseed oil. Values are shown as mean,* *n ≥ 9.*

(B)

(F)

(D)

(E)

(C)

(A)


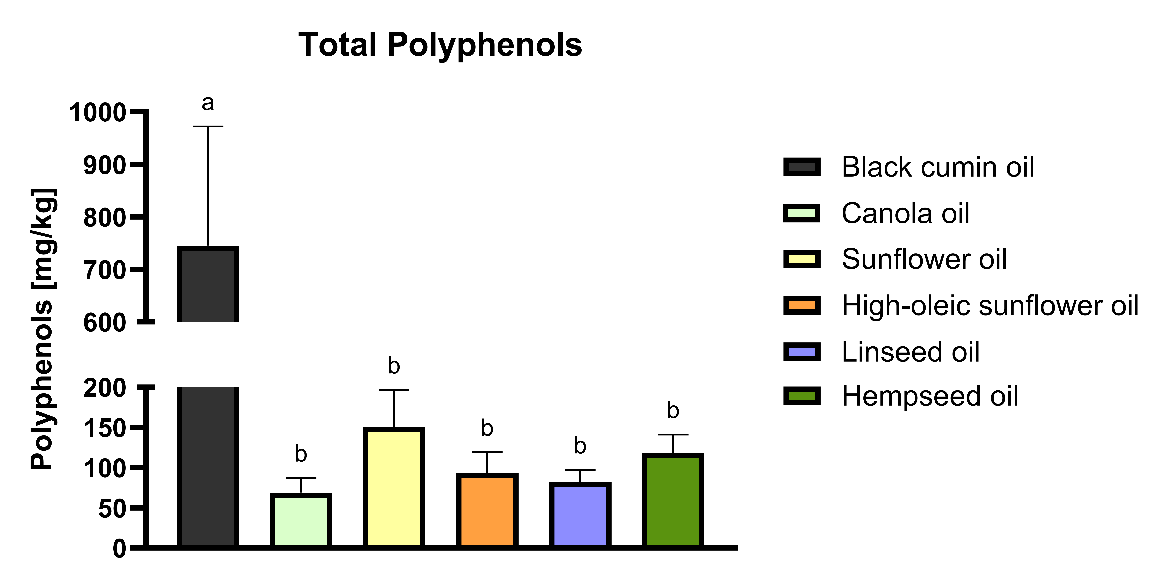


**Fig. S3:** Total polyphenol content in mg/kg oil in six cold-pressed oils at day 0 (T0) determined by LC-MS/MS. One-way ANOVA was conducted. Different lowercase letters indicate significant differences (p < 0.05, ANOVA with Tukey’s post hoc test). Values are shown as mean + SD, n ≥ 9


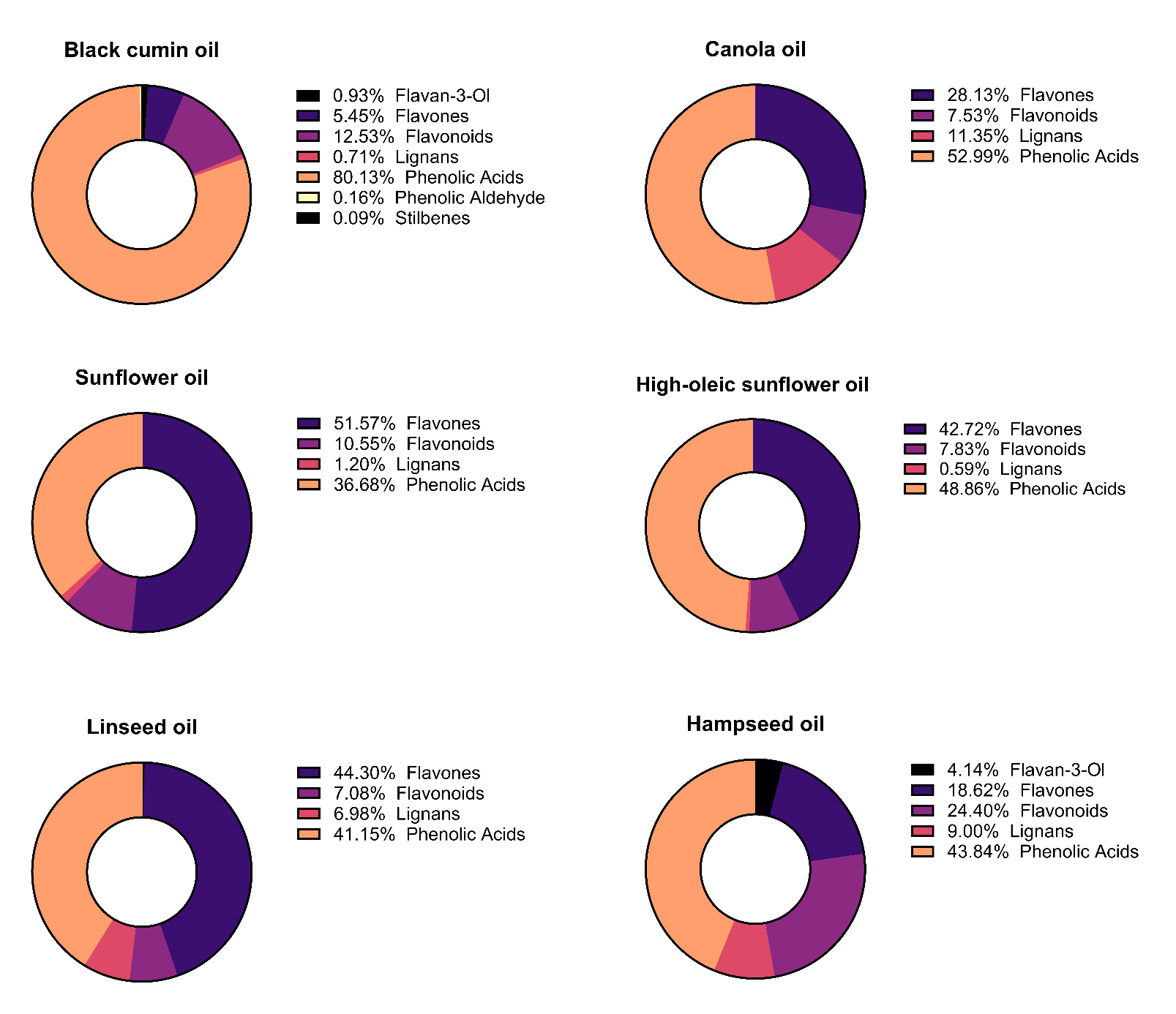


(F)

(B)

(D)

(E)

(C)

(A)

**Fig. S4:** Relative composition of polyphenol classes [%] in six cold-pressed oils at T0 (day 0), averaged across three suppliers. Data obtained via LC-MS/MS classification into phenolic acids, flavonoids, stilbenes, and lignans. Values are shown as relative mean of total polyphenol content, n ≥ 9.


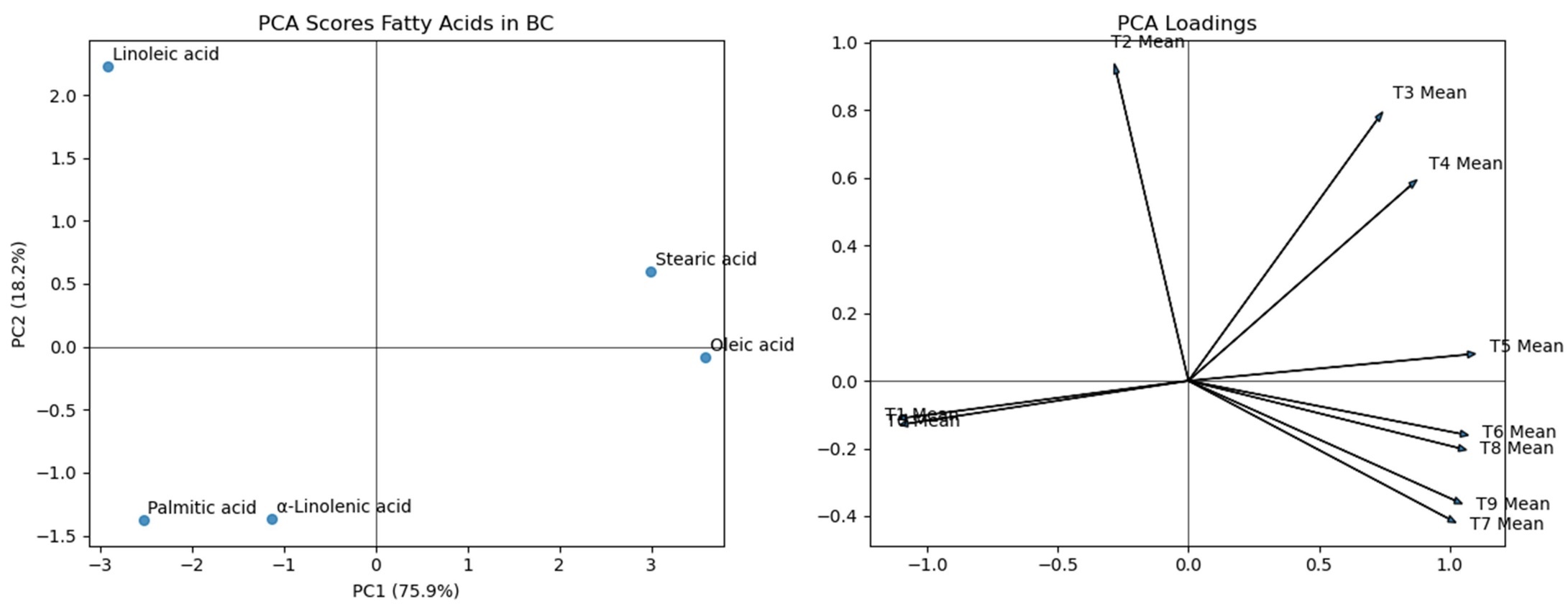


b)

a)

α-

**Figure S5**: a) Principal Component Analysis (PCA) of fatty acid profiles in black cumin oil (BC). Scores plot showing separation of fatty acids along the first two principal components. b) loadings plot illustrating the contribution of sampling time points (T0–T9, day 0 to 168) to the variance structure. PC1 and PC2 explain 75.9% and 18.2% of the total variance, respectively (cumulative 94.1%).

**Table S1**: Standard curves used for the fatty acid analysis by GC-FID. x = concentration [g/kg oil] and y = area under the curve (AuC) and coefficient of determination (R²).

| Standard | Formula | R^2^ | LOD [µg/g oil] | LOQ [µg/g oil] |
| --- | --- | --- | --- | --- |
| Methyl palmytate | y = 1.7026x - 0.1025 | 99.86 | 14 | 43 |
| Methyl stearate | y = 1.2663x - 0.0452 | 98.67 | 1.82 | 54 |
| Methyl oleate | y = 1.654x - 0.4769 | 99.63 | 84 | 255 |
| Methyl linoleate | y = 1.7386x - 0.0844 | 99.91 | 13 | 39 |
| Methyl linolenate | y = 1.3398x - 0.0057 | 99.90 | 1 | 4 |

**Table S2**: Standard curves from each of the representative standards used for quantification of polyphenolic substances identified with x = concentration [µg/g oil] and y = area under the curve (AuC) by LC-MS/MS with limit of detection (LOD) (signal-to-noise ratio of 3) and a limit of quantification (LOQ) (signal-to-noise ratio of 10) and coefficient of determination (R²).

| Standard | Formula | R^2^ | LOD [µg/g] | LOQ [µg/g] |
| --- | --- | --- | --- | --- |
| Kaempferol | y = 6976x - 7692 | 97.56 | 2.17 | 7.23 |
| Quercetin-3-glucoside | y = 44641x - 95622 | 98.27 | 1.82 | 6.07 |
| Naringenin | y = 131240x - 122238 | 98.73 | 1.56 | 5.20 |
| Apigenin | y = 185771x - 124518 | 99.87 | 0.46 | 1.54 |
| Epicatechin | y = 2059x + 32561 | 99.04 | 1.61 | 5.37 |
| Secoisolariciresinol | y = 771x + 545 | 99.58 | 0.52 | 1.73 |
| Caffeic acid | y = 96713x + 349596 | 99.83 | 1.31 | 4.39 |
| Ferulic acid | y = 190086x - 121795 | 98.16 | 1.88 | 6.27 |
| p-Coumaric acid | y = 123484x - 305192 | 99.13 | 1.29 | 4.29 |
| Gallic acid | y = 28929x + 61422 | 99.64 | 0.82 | 2.74 |
| Vanillic acid | y = 3721x - 1749 | 99.65 | 0.99 | 3.28 |
| 4-Hydroxybenzoic acid | y = 23581x + 116242 | 99.72 | 0.91 | 3.04 |
| Resveratrol | y = 1252x - 870 | 99.36 | 0.96 | 3.19 |
|  |  |  |  |  |

**Table S3**: Secondary lipid oxidation products used for quantification. Calibration curve equations are shown with coefficient of determination (R²).

| Standard | Formula | R^2^ |
| --- | --- | --- |
| Hexanal | y = 835429x + 5432321 | 95.81 |
| 2-pentanone | y = 3145202x - 4562391 | 98.41 |
| 2-heptanone | y = 1337482x + 2163811 | 98.32 |
| 1-hexanol | y = 1377884x + 2764936 | 91.56 |
|  |  |  |

**Table S4:** Transition list of all analyzed polyphenolic substances, grouped by class, including their precursor and product ion m/z values used for LC-MS/MS data acquisition in MRM mode.

| Polyphenol Class | Molecule Name | Precursor [m/z] | Product [m/z] |
| --- | --- | --- | --- |
| Phenolic Acids | 1,3-Dicaffeoylquinic acid | 515.0 | 353.0, 191.0, 171.0 |
| Phenolic Acids | 3,4-Dimethoxycinnamic acid | 207.1 | 207.1, 119.0, 103.0 |
| Phenolic Acids | 4-Hydroxybenzoic acid | 137.0 | 119.1, 108.0, 93.0, 59.2 |
| Phenolic Acids | 4-O-Caffeoylquinic acid | 353.1 | 191.0, 179.0, 173.0 |
| Flavones | Amentoflavone | 539.1 | 403.0, 377.1 |
| Flavonoids | Apigenin | 269.0 | 151.0, 117.0, 107.0 |
| Flavonoids | Astragalin | 447.4 | 284.0, 255.0, 227.0 |
| Phenolic Acids | Caffeic acid | 179.0 | 135.0, 107.0, 97.0 |
| Phenolic Acids | Caffeic acid hexoside | 341.1 | 179.0, 135.0, 103.0 |
| Flavonoids | Chalcone | 209.1 | 131.0, 103.0 |
| Phenolic Acids | Chlorogenic acid | 353.1 | 191.2, 179.0 |
| Phenolic Acids | cis Cinnamic acid | 149.1 | 131.0, 103.0 |
| Phenolic Acids | Coniferyl aldehyde | 177.1 | 162.0, 81.0 |
| Phenolic Acids | Coumaric acid hexoside | 325.1 | 205.0, 145.0, 117.0 |
| Phenolic Acids | Ellagic acid | 303.0 | 257.0, 229.0 |
| Flavan-3-Ol | Epicatechin | 289.1 | 245.1, 125.0, 109.0 |
| Flavonoids | Eriodictyol | 287.1 | 255.0, 151.0, 135.0 |
| Phenolic Acids | Ferulic acid | 195.0 | 177.0, 117.0, 89.0 |
| Phenolic Acids | Gallic acid | 169.0 | 125.0, 96.6, 79.1 |
| Flavan-3-Ol | Gallocatechol | 305.1 | 137.0, 125.0 |
| Flavones | Herbacetin | 303.0 | 169.0, 133.0, 121.0 |
| Flavonoids | Hesperetin | 303.3 | 177.0, 153.0 |
| Flavonoids | Hesperidin | 609.2 | 301.1 |
| Phenolic Acids | Hydroxyferulic acid | 209.0 | 165.0, 121.0 |
| Phenolic Acids | Isoferulic acid | 193.0 | 178.3, 133.8 |
| Flavonoids | Isorhamnetin | 315.0 | 151.0, 107.0 |
| Flavonoids | Isorhamnetin 3-rutinoside | 625.2 | 302.0 |
| Flavonoids | Isorhamnetin-3-O-glucoside | 477.1 | 314.0, 271.0, 243.0 |
| Flavonoids | Kaempferol | 285.1 | 257.0, 229.0 |
| Flavonoids | Kaempferol-3-O-arabinoside | 417.0 | 284.0, 255.0, 227.0 |
| Flavonoids | Kaempferol-3-O-glucoside | 447.1 | 285.0, 247.1 |
| Flavonoids | Kaempferol-3-O-glucuronic acid | 461.1 | 285.0, 229.0 |
| Flavonoids | Kaempferol-3-O-hexoxyl-hexoside | 609.1 | 284.0 |
| Lignans | Lariciresinol | 359.1 | 330.0, 160.1 |
| Flavonoids | Luteolin | 285.0 | 151.0, 133.0, 107.0 |
| Flavonoids | Luteolin-7-O-glucoside | 447.1 | 285.0, 151.0 |
| Lignans | Matairesinol | 357.1 | 137.0, 122.0, 83.0 |
| Flavonoids | Naringenin | 271.2 | 151.0, 119.0 |
| Flavonoids | Naringin | 579.2 | 459.0, 271.1, 151.0 |
| Phenolic Acids | p-Coumaric acid | 165.0 | 147.0, 119.0, 91.0 |
| Lignans | Pinoresinol | 357.1 | 151.0, 136.0 |
| Phenolic Acids | Protocatechuic acid | 153.0 | 109.0, 81.0 |
| Stilbenes | Pterostilbene | 255.1 | 195.0, 169.0 |
| Flavonoids | Quercetin | 301.0 | 151.0, 121.0, 93.0 |
| Flavonoids | Quercetin-3-(sinapoyl-diglucoside)-7-glucoside | 625.1 | 463.0, 301.0 |
| Flavonoids | Quercetin-3-O-glucoronic acid | 477.1 | 301.0, 151.0, 107.0 |
| Flavonoids | Quercetin-3-O-glucoside | 463.0 | 301.0, 271.0 |
| Phenolic Acids | Quinic acid | 191.0 | 93.0, 85.0 |
| Stilbenes | Resveratrol | 227.0 | 185.0, 143.0 |
| Flavonoids | Rutin | 609.0 | 300.0, 271.0 |
| Lignans | Secoisolariciresinol | 363.2 | 327.2, 137.1 |
| Lignans | Secoisolariciresinol diglucoside | 685.0 | 523.0, 361.0 |
| Phenolic Acids | Sinapic acid | 223.0 | 163.0, 149.0 |
| Phenolic Acids | Syringic acid | 197.0 | 120.9, 105.8 |
| Phenolic Acids | Syringic aldehyde | 181.0 | 151.0, 123.0 |
| Phenolic Acids | Vanillic acid | 167.0 | 152.0, 108.0 |
| Phenolic Aldehyde | Vanillin | 151.0 | 136.0, 92.0 |

***Table S5:*** *GC-FID analysis of fatty acids in six cold-pressed oils during storage. Data given as mean ± standard deviation (SD) in mg/g oil, n=9.*

| Black Cumin Oil |  |  |  |  |  |  |  |  |  |  |
| --- | --- | --- | --- | --- | --- | --- | --- | --- | --- | --- |
| Substance | Day 0 | Day 3 | Day 7 | Day 14 | Day 28 | Day 42 | Day 56 | Day 84 | Day 112 | Day 168 |
| Palmitic acid | 112.64 ± 7.85 | 109.36 ± 4.53 | 78.95 ± 1.54 | 59.81 ± 1.01 | 48.48 ± 1.01 | 44.51 ± 0.94 | 44.31 ± 1.04 | 46.38 ± 0.41 | 44.51 ± 0.91 | 41.88 ± 0.35 |
| Stearic acid | 16.78 ± 1.01 | 16.29 ± 4.33 | 23.94 ± 0.18 | 19.02 ± 0.29 | 15.67 ± 0.29 | 14.46 ± 0.30 | 13.82 ± 0.30 | 15.06 ± 0.17 | 14.54 ± 0.31 | 13.61 ± 0.11 |
| Oleic acid | 118.26 ± 6.03 | 114.82 ± 28.99 | 147.19 ± 1.05 | 136.70 ± 2.06 | 112.53 ± 2.05 | 103.97 ± 1.76 | 101.12 ± 1.52 | 104.65 ± 1.00 | 97.87 ± 1.98 | 95.03 ± 0.68 |
| Linoleic acid | 534.15 ± 33.58 | 514.97 ± 27.16 | 424.14 ± 11.96 | 320.15 ± 5.25 | 257.21 ± 5.22 | 216.76 ± 3.99 | 196.61 ± 3.25 | 197.91 ± 2.30 | 199.23 ± 4.63 | 178.92 ± 1.67 |
| α-Linolenic acid | 5.14 ± 0.34 | 4.80 ± 0.93 | 3.71 ± 0.08 | 2.99 ± 0.05 | 2.56 ± 0.08 | 2.34 ± 0.07 | 2.14 ± 0.07 | 2.41 ± 0.08 | 2.25 ± 0.04 | 2.13 ± 0.06 |
| Canola Oil |  |  |  |  |  |  |  |  |  |  |
| Palmitic acid | 35.24 ± 0.90 | 44.99 ± 1.56 | 33.29 ± 0.18 | 30.82 ± 0.35 | 27.37 ± 1.15 | 26.89 ± 0.55 | 21.91 ± 0.76 | 24.37 ± 0.37 | 22.15 ± 0.77 | 19.55 ± 0.14 |
| Stearic acid | 12.22 ± 0.44 | 11.44 ± 0.28 | 18.18 ± 0.11 | 17.02 ± 0.17 | 16.59 ± 0.91 | 14.00 ± 0.26 | 11.62 ± 0.43 | 13.27 ± 0.28 | 12.20 ± 0.48 | 10.48 ± 0.06 |
| Oleic acid | 352.43 ± 14.69 | 325.08 ± 8.70 | 349.89 ± 14.12 | 352.38 ± 5.17 | 318.84 ± 15.62 | 310.51 ± 8.26 | 287.82 ± 11.49 | 297.92 ± 6.88 | 292.16 ± 13.11 | 252.86 ± 2.10 |
| Linoleic acid | 164.31 ± 6.58 | 151.14 ± 6.54 | 126.67 ± 4.75 | 113.04 ± 6.19 | 100.01 ± 6.08 | 77.72 ± 2.61 | 70.74 ± 2.96 | 68.38 ± 1.79 | 73.88 ± 3.27 | 63.29 ± 0.56 |
| α-Linolenic acid | 71.73 ± 3.27 | 67.98 ± 2.11 | 59.63 ± 3.06 | 55.63 ± 1.02 | 50.57 ± 1.38 | 40.11 ± 2.12 | 34.91 ± 1.98 | 33.04 ± 1.12 | 37.12 ± 1.26 | 31.59 ± 0.33 |
| Sunflower Oil |  |  |  |  |  |  |  |  |  |  |
| Palmitic acid | 37.47 ± 2.43 | 44.10 ± 1.82 | 41.99 ± 0.30 | 31.93 ± 0.36 | 28.76 ± 6.96 | 28.63 ± 0.25 | 29.81 ± 0.56 | 26.87 ± 0.18 | 25.95 ± 0.59 | 23.95 ± 0.29 |
| Stearic acid | 20.32 ± 0.68 | 20.17 ± 0.58 | 28.08 ± 0.31 | 27.97 ± 0.29 | 24.92 ± 0.19 | 22.45 ± 0.42 | 19.22 ± 0.46 | 20.89 ± 0.55 | 21.17 ± 0.47 | 18.71 ± 0.29 |
| Oleic acid | 193.32 ± 8.27 | 177.96 ± 4.78 | 168.47 ± 3.68 | 164.67 ± 6.84 | 176.10 ± 6.39 | 155.23 ± 5.06 | 166.39 ± 9.52 | 161.59 ± 3.75 | 159.52 ± 3.93 | 146.91 ± 1.90 |
| Linoleic acid | 439.66 ± 16.27 | 419.57 ± 14.41 | 385.22 ± 5.99 | 348.76 ± 6.18 | 295.52 ± 7.94 | 262.23 ± 6.86 | 224.52 ± 7.13 | 202.68 ± 3.79 | 206.41 ± 4.82 | 185.77 ± 2.85 |
| α-Linolenic acid | 0.95 ± 0.04 | 0.92 ± 0.04 | 0.87 ± 0.06 | 0.80 ± 0.05 | 0.71 ± 0.03 | 0.69 ± 0.06 | 0.70 ± 0.04 | 0.70 ± 0.04 | 0.58 ± 0.05 | 0.59 ± 0.06 |
| High-Oleic Sunflower Oil |  |  |  |  |  |  |  |  |  |  |
| Palmitic acid | 42.87 ± 2.13 | 38.59 ± 3.54 | 26.40 ± 0.13 | 24.22 ± 0.58 | 21.91 ± 1.16 | 21.06 ± 1.34 | 19.87 ± 2.81 | 17.80 ± 0.18 | 17.76 ± 0.24 | 15.34 ± 0.22 |
| Stearic acid | 23.78 ± 1.17 | 19.83 ± 1.75 | 31.01 ± 0.15 | 26.52 ± 4.68 | 23.51 ± 1.48 | 21.20 ± 1.65 | 18.42 ± 0.25 | 18.83 ± 2.90 | 19.97 ± 0.34 | 17.10 ± 0.28 |
| Oleic acid | 580.86 ± 27.08 | 545.72 ± 39.13 | 537.85 ± 13.86 | 489.56 ± 11.07 | 428.21 ± 27.33 | 419.46 ± 24.83 | 355.11 ± 15.28 | 323.77 ± 5.21 | 391.68 ± 6.56 | 344.08 ± 6.55 |
| Linoleic acid | 50.39 ± 2.87 | 48.42 ± 4.55 | 35.39 ± 1.38 | 33.05 ± 1.51 | 27.38 ± 1.49 | 24.63 ± 3.34 | 21.13 ± 0.77 | 18.19 ± 0.25 | 22.04 ± 0.34 | 18.82 ± 0.30 |
| α-Linolenic acid | 1.13 ± 0.11 | 0.99 ± 0.10 | 0.90 ± 0.04 | 0.88 ± 0.05 | 0.75 ± 0.03 | 0.79 ± 0.07 | 0.65 ± 0.04 | 0.70 ± 0.11 | 0.67 ± 0.04 | 0.60 ± 0.03 |
| Linseed Oil |  |  |  |  |  |  |  |  |  |  |
| Palmitic acid | 37.53 ± 3.01 | 34.21 ± 3.64 | 38.86 ± 0.18 | 37.44 ± 0.25 | 32.06 ± 1.51 | 27.16 ± 0.03 | 25.42 ± 0.08 | 27.22 ± 0.15 | 24.60 ± 1.12 | 22.38 ± 0.19 |
| Stearic acid | 21.51 ± 1.05 | 21.82 ± 1.36 | 27.85 ± 0.20 | 30.50 ± 0.24 | 28.92 ± 1.29 | 24.64 ± 0.03 | 21.09 ± 0.09 | 23.08 ± 0.24 | 21.58 ± 1.11 | 19.19 ± 0.20 |
| Oleic acid | 94.30 ± 4.15 | 95.59 ± 5.69 | 114.17 ± 3.27 | 111.97 ± 4.24 | 119.95 ± 5.31 | 108.30 ± 3.42 | 97.20 ± 4.67 | 102.85 ± 3.15 | 91.72 ± 3.81 | 83.70 ± 0.77 |
| Linoleic acid | 130.10 ± 10.80 | 132.50 ± 11.43 | 120.39 ± 8.91 | 111.01 ± 7.32 | 93.66 ± 6.34 | 76.38 ± 4.58 | 68.07 ± 2.36 | 72.82 ± 3.16 | 67.65 ± 3.57 | 60.35 ± 0.71 |
| α-Linolenic acid | 535.02 ± 32.93 | 530.08 ± 39.22 | 494.73 ± 23.09 | 449.47 ± 16.57 | 369.01 ± 23.22 | 347.66 ± 8.32 | 279.03 ± 8.67 | 281.46 ± 6.76 | 264.78 ± 15.00 | 236.66 ± 3.19 |
| Hempseed Oil |  |  |  |  |  |  |  |  |  |  |
| Palmitic acid | 41.16 ± 1.85 | 49.04 ± 3.13 | 43.91 ± 0.56 | 36.48 ± 1.18 | 34.54 ± 0.24 | 33.81 ± 0.02 | 28.50 ± 0.22 | 31.63 ± 0.59 | 27.91 ± 1.07 | 24.78 ± 0.36 |
| Stearic acid | 11.17 ± 0.51 | 13.16 ± 0.58 | 21.44 ± 0.29 | 20.71 ± 0.67 | 16.38 ± 0.16 | 14.19 ± 0.25 | 15.43 ± 0.33 | 17.68 ± 0.43 | 15.94 ± 0.71 | 13.74 ± 0.27 |
| Oleic acid | 72.40 ± 1.51 | 69.77 ± 1.91 | 73.67 ± 1.98 | 73.63 ± 3.30 | 76.97 ± 1.92 | 73.85 ± 0.65 | 69.35 ± 2.76 | 73.86 ± 1.35 | 67.75 ± 2.14 | 58.23 ± 0.80 |
| Linoleic acid | 416.47 ± 18.07 | 402.87 ± 24.67 | 376.82 ± 9.30 | 331.96 ± 13.31 | 297.54 ± 6.22 | 264.84 ± 7.43 | 213.02 ± 3.29 | 214.80 ± 5.74 | 209.86 ± 9.73 | 181.57 ± 3.20 |
| α-Linolenic acid | 152.68 ± 8.08 | 151.08 ± 8.73 | 142.51 ± 5.63 | 120.89 ± 7.22 | 110.84 ± 3.33 | 91.21 ± 2.90 | 81.41 ± 2.16 | 83.71 ± 2.64 | 82.77 ± 3.94 | 71.34 ± 1.33 |

***Table S6:*** *Targeted LC-MS/MS analysis of polyphenols in six cold-pressed oils. Data given as mean ± standard deviation (SD) in ppm, n=9.*

| Black Cumin Oil |  |  |  |  |  |  |  |  |  |  |  |
| --- | --- | --- | --- | --- | --- | --- | --- | --- | --- | --- | --- |
| Polyphenol Class | Substance | Day 0 | Day 3 | Day 7 | Day 14 | Day 28 | Day 42 | Day 56 | Day 84 | Day 112 | Day 168 |
| Flavan-3-Ol | Epicatechin | 5.64 ± 1.74 | 1.23 ± 0.36 | 0.80 ± 0.19 | 1.90 ± 0.63 | 1.47 ± 0.38 | 1.37 ± 0.32 | 1.72 ± 0.57 | 0.70 ± 0.04 | 1.94 ± 0.15 | 2.55 ± 1.00 |
| Flavan-3-Ol | Gallocatechol | 1.31 ± 0.28 | 0.20 ± 0.02 | LOQ | 0.28 ± 0.02 | 0.29 ± 0.02 | 0.29 ± 0.01 | 0.34 ± 0.13 | 0.38 ± 0.02 | 0.31 ± 0.04 | LOQ |
| Flavones | Amentoflavone | 0.11 ± 0.03 | 0.22 ± 0.08 | LOQ | LOQ | LOQ | LOQ | LOQ | LOD | LOD | LOD |
| Flavones | Herbacetin | 40.63 ± 8.12 | 13.22 ± 3.84 | 11.32 ± 1.54 | 5.19 ± 1.87 | 4.38 ± 0.67 | 6.08 ± 1.42 | 6.53 ± 1.15 | 3.73 ± 1.07 | 0.58 ± 0.29 | 0.54 ± 0.22 |
| Flavonoids | Apigenin | 2.48 ± 1.04 | 0.22 ± 0.11 | 0.11 ± 0.05 | 0.29 ± 0.13 | LOQ | LOD | LOD | LOD | 0.34 ± 0.06 | LOQ |
| Flavonoids | Chalcone | 0.29 ± 0.10 | LOQ | LOQ | LOD | LOD | LOD | LOD | LOD | LOD | LOD |
| Flavonoids | Eriodictyol | 1.47 ± 0.39 | 0.28 ± 0.07 | 0.30 ± 0.04 | 0.26 ± 0.01 | 0.24 ± 0.08 | LOQ | LOQ | LOQ | LOD | LOD |
| Flavonoids | Hesperetin | 7.52 ± 2.01 | 0.93 ± 0.21 | 1.13 ± 0.62 | 1.85 ± 1.02 | 0.63 ± 0.18 | LOD | LOQ | LOQ | LOD | LOD |
| Flavonoids | Hesperidin | 0.28 ± 0.06 | LOQ | LOQ | LOQ | LOD | LOD | LOD | LOD | LOD | LOD |
| Flavonoids | Isorhamnetin | 10.92 ± 3.24 | 1.46 ± 0.54 | 1.22 ± 0.34 | 0.96 ± 0.53 | 0.58 ± 0.24 | 0.48 ± 0.11 | 0.42 ± 0.07 | 0.41 ± 0.20 | LOD | LOD |
| Flavonoids | Isorhamnetin 3-rutinoside | 4.63 ± 1.34 | 1.13 ± 0.43 | 0.92 ± 0.34 | 0.29 ± 0.03 | 0.27 ± 0.05 | 0.34 ± 0.09 | LOQ | LQO | LOD | LOD |
| Flavonoids | Isorhamnetin-3-O-glucoside | 3.38 ± 0.46 | 0.47 ± 0.16 | 0.23 ± 0.02 | 0.89 ± 0.16 | 0.44 ± 0.15 | LOQ | LOQ | 0.50 ± 0.27 | 0.34 ± 0.01 | 0.32 ± 0.11 |
| Flavonoids | Kaempferol | 9.71 ± 1.33 | 0.63 ± 0.14 | 0.74 ± 0.20 | 2.01 ± 1.37 | 0.53 ± 0.18 | 0.58 ± 0.15 | LOQ | 0.74 ± 0.22 | 0.48 ± 0.11 | 0.40 ± 0.07 |
| Flavonoids | Kaempferol-3-O-arabinoside | 0.89 ± 0.18 | 0.38 ± 0.14 | 0.23 ± 0.07 | 2.23 ± 0.06 | 0.53 ± 0.22 | 0.54 ± 0.22 | 1.13 ± 0.38 | 0.82 ± 0.39 | 0.46 ± 0.09 | 0.43 ± 0.19 |
| Flavonoids | Kaempferol-3-O-glucoside (Astragalin) | 22.60 ± 3.60 | 6.65 ± 1.82 | 6.90 ± 1.58 | 12.40 ± 6.24 | 3.12 ± 1.07 | 2.67 ± 0.86 | 2.62 ± 0.68 | 2.93 ± 1.37 | 0.46 ± 0.17 | 0.41 ± 0.24 |
| Flavonoids | Kaempferol-3-O-glucuronic acid | 13.76 ± 3.68 | 2.74 ± 0.88 | 2.25 ± 0.60 | 1.81 ± 0.79 | 1.48 ± 0.60 | 1.05 ± 0.36 | 0.19 ± 0.07 | 0.46 ± 0.04 | 0.51 ± 0.19 | 0.46 ± 0.15 |
| Flavonoids | Kaempferol-3-O-hexoxyl-hexoside | 1.01 ± 0.27 | 0.37 ± 0.11 | 0.17 ± 0.04 | 0.42 ± 0.16 | 0.20 ± 0.03 | 0.17 ± 0.02 | 0.46 ± 0.01 | LOQ | 0.38 ± 0.02 | 0.46 ± 0.19 |
| Flavonoids | Luteolin | 0.65 ± 0.37 | 0.18 ± 0.03 | LOQ | LOQ | LOD | LOD | LOD | LOD | LOD | LOD |
| Flavonoids | Luteolin-7-O-glucoside | 0.88 ± 0.28 | LOD | LOD | LOD | LOD | LOD | LOD | LOD | LOD | LOD |
| Flavonoids | Naringenin | 1.74 ± 0.26 | 0.34 ± 0.08 | 0.16 ± 0.03 | 0.20 ± 0.02 | 0.15 ± 0.03 | 0.16 ± 0.06 | LOD | LOD | LOD | LOD |
| Flavonoids | Naringin | 1.61 ± 0.45 | 0.20 ± 0.10 | 0.06 ± 0.02 | 0.23 ± 0.09 | 0.16 ± 0.01 | 0.17 ± 0.02 | 0.19 ± 0.06 | LOD | LOD | LOD |
| Flavonoids | Quercetin | 2.51 ± 0.35 | 0.51 ± 0.10 | 0.42 ± 0.10 | 0.70 ± 0.30 | 0.65 ± 0.20 | 0.43 ± 0.05 | 0.44 ± 0.11 | 0.44 ± 0.08 | 0.24 ± 0.09 | LOD |
| Flavonoids | quercetin-3-(sinapoyl-diglucoside)-7-glucoside | 0.63 ± 0.18 | 0.32 ± 0.05 | 0.33 ± 0.05 | 0.32 ± 0.03 | 0.35 ± 0.09 | 0.23 ± 0.04 | 0.32 ± 0.10 | 0.26 ± 0.03 | 0.22 ± 0.02 | 0.29 ± 0.04 |
| Flavonoids | Quercetin-3-O-glucoronic acid | 3.28 ± 0.96 | 0.58 ± 0.17 | 0.48 ± 0.05 | 4.34 ± 0.82 | 0.48 ± 0.13 | 0.28 ± 0.03 | LOQ | 0.25 ± 0.03 | 0.28 ± 0.11 | LOQ |
| Flavonoids | Quercetin-3-O-glucoside | 2.22 ± 0.45 | 0.32 ± 0.05 | 0.21 ± 0.07 | 0.44 ± 0.11 | 0.31 ± 0.11 | LOQ | 0.27 ± 0.07 | LOD | 0.32 ± 0.07 | LOD |
| Lignans | Lariciresinol | 0.27 ± 0.09 | 0.18 ± 0.01 | 0.08 ± 0.03 | LOD | LOD | LOD | LOD | LOD | LOD | LOD |
| Lignans | Matairesinol | 1.51 ± 0.19 | 0.72 ± 0.28 | 0.28 ± 0.06 | 0.35 ± 0.13 | 0.39 ± 0.13 | 0.30 ± 0.08 | 0.20 ± 0.07 | 0.23 ± 0.07 | LOD | LOD |
| Lignans | Pinoresinol | 1.85 ± 0.14 | 1.43 ± 0.41 | 1.07 ± 0.56 | 1.09 ± 0.60 | 1.01 ± 0.39 | 1.12 ± 0.52 | 0.64 ± 0.18 | 0.68 ± 0.38 | 0.29 ± 0.08 | 0.18 ± 0.08 |
| Lignans | Secoisolariciresinol | 1.59 ± 0.46 | 0.66 ± 0.28 | 0.29 ± 0.10 | 0.35 ± 0.03 | LOD | LOD | LOD | LOD | LOD | LOD |
| Lignans | Secoisolariciresinol diglucoside | LOQ | LOQ | LOD | LOD | LOD | LOD | LOD | LOD | LOD | LOD |
| Phenolic Acids | 3,4-Dimethoxycinnamic acid | LOQ | LOQ | 0.26 ± 0.05 | 0.32 ± 0.04 | 1.09 ± 0.26 | 0.76 ± 0.06 | 0.64 ± 0.03 | 0.49 ± 0.06 | LOD | LOD |
| Phenolic Acids | 4-Hydroxybenzoic acid | 13.07 ± 1.18 | 2.29 ± 0.58 | 1.73 ± 0.37 | 1.26 ± 0.59 | 0.75 ± 0.16 | 0.79 ± 0.17 | 0.64 ± 0.19 | 0.58 ± 0.12 | 2.30 ± 0.18 | 1.07 ± 0.63 |
| Phenolic Acids | 4-O-Caffeoylquinic acid | LOQ | LOQ | LOQ | LOQ | LOD | LOQ | LOD | LOD | LOD | LOD |
| Phenolic Acids | Caffeic acid | 2.74 ± 0.58 | 1.88 ± 1.05 | LOQ | LOQ | LOQ | LOQ | LOQ | LOQ | LOD | LOD |
| Phenolic Acids | Caffeic acid hexoside | 2.73 ± 0.13 | 1.03 ± 0.26 | 0.41 ± 0.07 | 0.67 ± 0.34 | 0.70 ± 0.26 | 0.45 ± 0.13 | 0.78 ± 0.30 | 0.49 ± 0.16 | LOD | LOD |
| Phenolic Acids | Chlorogenic acid | LOQ | LOQ | LOQ | LOQ | LOQ | LOQ | LOD | LOQ | LOQ | LOD |
| Phenolic Acids | cis Cinnamic acid | 11.31 ± 1.95 | 6.30 ± 2.21 | 1.96 ± 0.47 | 1.61 ± 0.80 | 0.94 ± 0.26 | 0.88 ± 0.22 | 0.87 ± 0.19 | 0.73 ± 0.23 | LOD | LOD |
| Phenolic Acids | Coniferyl aldehyde | 0.04 ± 0.00 | 0.02 ± 0.00 | 0.07 ± 0.04 | 0.10 ± 0.06 | 0.15 ± 0.05 | 0.24 ± 0.11 | 0.28 ± 0.13 | LOQ | LOQ | LOQ |
| Phenolic Acids | Ferulic acid | 2.53 ± 0.49 | 0.83 ± 0.15 | 0.24 ± 0.05 | LOQ | LOQ | LOQ | LOQ | LOQ | LOD | LOD |
| Phenolic Acids | Gallic acid | 0.86 ± 0.19 | 0.37 ± 0.15 | 0.38 ± 0.08 | 0.63 ± 0.31 | 0.24 ± 0.08 | 0.54 ± 0.19 | 0.46 ± 0.21 | 0.43 ± 0.12 | 1.77 ± 0.44 | LOQ |
| Phenolic Acids | Hydroxyferulic acid | 1.46 ± 0.72 | 0.80 ± 0.22 | 0.26 ± 0.07 | LOQ | LOQ | LOQ | LOQ | LOD | LOD | LOD |
| Phenolic Acids | p-Coumaric acid | 538.20 ± 68.93 | 248.12 ± 66.03 | 63.40 ± 9.22 | 46.57 ± 19.22 | 29.86 ± 7.15 | 34.08 ± 7.72 | 41.69 ± 10.37 | 33.64 ± 8.24 | 22.02 ± 8.52 | 13.22 ± 8.78 |
| Phenolic Acids | Quinic acid | 25.32 ± 1.23 | 23.84 ± 1.33 | 23.55 ± 1.07 | 21.08 ± 7.08 | 20.20 ± 1.49 | 19.21 ± 1.52 | 16.64 ± 0.89 | 14.12 ± 0.91 | 15.71 ± 5.49 | 2.42 ± 1.77 |
| Phenolic Acids | Sinapic acid | 0.48 ± 0.10 | 0.11 ± 0.01 | 0.07 ± 0.02 | 2.85 ± 1.93 | 0.31 ± 0.10 | 0.39 ± 0.22 | 0.62 ± 0.28 | 0.86 ± 0.45 | 2.65 ± 0.18 | 2.41 ± 1.77 |
| Phenolic Acids | Syringic aldehyde | 0.08 ± 0.01 | 0.04 ± 0.01 | 0.01 ± 0.00 | 0.04 ± 0.00 | LOD | LOD | LOD | LOD | 0.13 ± 0.07 | LOD |
| Phenolic Aldehyde | Vanillin | 12.25 ± 1.33 | 2.18 ± 1.09 | 0.43 ± 0.11 | 0.38 ± 0.14 | 0.83 ± 0.02 | 0.76 ± 0.07 | 0.34 ± 0.05 | 0.45 ± 0.07 | 0.31 ± 0.12 | 1.50 ± 0.81 |
| Stilbenes | Pterostilbene | 3.81 ± 0.91 | 2.50 ± 0.79 | 2.16 ± 0.23 | 3.07 ± 0.45 | 2.46 ± 0.41 | 3.06 ± 0.39 | 3.08 ± 0.84 | 1.19 ± 0.06 | 3.12 ± 0.95 | 3.00 ± 0.59 |
| Stilbenes | Resveratrol | 2.63 ± 0.70 | 1.96 ± 0.74 | 1.79 ± 0.65 | 2.29 ± 0.35 | 1.41 ± 0.52 | 1.83 ± 0.67 | 1.54 ± 0.09 | 1.85 ± 0.21 | 1.31 ± 0.23 | 1.99 ± 0.43 |
|  | 5-Ethyl-2(5)-furanone | 0.30 ± 0.09 | 5.08 ± 1.68 | 0.03 ± 0.01 |  |  |  |  |  |  |  |
| Canola Oil |  |  |  |  |  |  |  |  |  |  |  |
| Flavones | Amentoflavone | 0.41± 0.04 | 0.60± 0.23 | 0.31± 0.12 | 0.39± 0.14 | 0.20± 0.02 | 0.44± 0.08 | 0.47± 0.14 | 0.42± 0.08 | LOD | LOQ |
| Flavones | Herbacetin | 19.11± 7.09 | 6.32± 2.27 | 4.69± 1.21 | 3.60± 1.06 | 2.85± 1.73 | 2.61± 0.42 | 3.17± 0.66 | 2.56± 0.21 | 1.57± 0.30 | 1.41± 0.51 |
| Flavonoids | Apigenin | LOQ | LOQ | LOQ | LOQ | LOD | LOD | LOD | LOD | LOD | LOD |
| Flavonoids | Chalcone | 0.06± 0.01 | 0.05± 0.01 | LOD | LOD | LOD | LOD | LOD | LOD | LOD | LOD |
| Flavonoids | Eriodictyol | 0.57± 0.16 | LOQ | 0.45± 0.05 | 0.49± 0.17 | LOQ | LOQ | LOQ | 0.53± 0.10 | LOD | LOD |
| Flavonoids | Hesperetin | 1.43± 0.39 | 0.18± 0.07 | 0.22± 0.05 | 0.63± 0.18 | 0.46± 0.28 | 0.17± 0.00 | 0.09± 0.03 | 0.18± 0.00 | 0.18± 0.02 | LOD |
| Flavonoids | Hesperidin | LOQ | 0.17± 0.02 | 0.18± 0.02 | LOQ | 0.26± 0.13 | LOQ | LOQ | LOQ | LOQ | LOQ |
| Flavonoids | Isorhamnetin | LOQ | 0.14± 0.05 | 0.17± 0.05 | 0.18± 0.02 | 0.25± 0.14 | LOQ | LOQ | LOQ | LOD | LOD |
| Flavonoids | Isorhamnetin 3-Rutinoside | 1.08± 0.13 | 0.29± 0.03 | 0.38± 0.04 | 0.29± 0.09 | 0.62± 0.05 | LOQ | 0.29± 0.05 | LOQ | LOD | LOQ |
| Flavonoids | Kaempferol | 1.14± 0.25 | 0.48± 0.05 | 0.36± 0.10 | 0.57± 0.19 | 0.58± 0.33 | LOQ | LOQ | 0.33± 0.08 | 0.43± 0.13 | LOQ |
| Flavonoids | Naringenin | 0.22± 0.04 | LOQ | LOQ | 0.42± 0.05 | LOQ | LOQ | LOQ | LOQ | LOD | LOD |
| Flavonoids | Naringin | LOQ | LOQ | LOQ | LOQ | LOQ | LOQ | LOD | LOD | LOD | LOD |
| Flavonoids | Quercetin | 0.52± 0.10 | 0.26± 0.09 | 0.48± 0.11 | LOQ | 0.76± 0.34 | LOQ | LOQ | LOQ | LOQ | LOQ |
| Lignans | Lariciresinol | 4.97± 1.93 | 3.45± 1.59 | LOQ | LOQ | 1.02± 0.98 | 0.99± 0.47 | 0.56± 0.32 | 1.05± 0.59 | LOQ | LOQ |
| Lignans | Matairesinol | 1.59± 0.33 | 0.53± 0.17 | 0.43± 0.14 | 0.83± 0.26 | 0.94± 0.60 | LOQ | LOQ | LOQ | LOD | LOD |
| Lignans | Pinoresinol | 1.05± 0.14 | 0.33± 0.07 | LOQ | 0.84± 0.34 | LOQ | LOQ | LOQ | LOQ | LOD | LOD |
| Lignans | Secoisolariciresinol | LOQ | LOQ | LOQ | LOD | LOD | LOD | LOD | LOD | LOD | LOD |
| Phenolic Acids | 3,4-Dimethoxycinnamic acid | 0.21± 0.05 | 0.25± 0.03 | 0.31± 0.03 | 0.67± 0.18 | 1.51± 0.94 | 0.73± 0.08 | 0.66± 0.03 | 0.69± 0.04 | LOQ | LOD |
| Phenolic Acids | 4-Hydroxybenzoic acid | 0.74± 0.17 | LOQ | LOQ | 0.69± 0.18 | 0.51± 0.27 | LOQ | 0.24± 0.07 | LOQ | LOQ | LOD |
| Phenolic Acids | 4-O-Caffeoylquinic acid | 0.34± 0.02 | 0.38± 0.05 | 0.39± 0.05 | 0.49± 0.15 | 1.01± 0.23 | 0.30± 0.09 | 0.40± 0.14 | 0.39± 0.11 | LOQ | LOQ |
| Phenolic Acids | Caffeic acid | 0.24± 0.01 | 0.25± 0.02 | 0.18± 0.05 | 0.63± 0.15 | 0.53± 0.11 | LOQ | LOQ | 0.48± 0.08 | 0.39± 0.13 | 0.38± 0.17 |
| Phenolic Acids | Caffeic acid hexoside | 0.77± 0.12 | 0.37± 0.06 | 0.22± 0.03 | 0.54± 0.20 | 0.51± 0.30 | 0.03± 0.01 | 0.10± 0.03 | 0.12± 0.03 | LOD | LOD |
| Phenolic Acids | Chlorogenic acid | 0.22± 0.11 | 0.03± 0.00 | 0.05± 0.01 | 0.08± 0.03 | 0.23± 0.02 | LOD | LOD | LOD | LOD | LOD |
| Phenolic Acids | cis Cinnamic acid | 0.53± 0.16 | 0.08± 0.03 | 0.03± 0.00 | 0.37± 0.14 | 0.10± 0.08 | LOD | LOD | LOD | LOD | LOD |
| Phenolic Acids | Coumaric acid hexoside | LOQ | LOQ | LOQ | LOD | LOD | LOD | LOD | LOD | LOD | LOD |
| Phenolic Acids | Ellagic acid | 0.94± 0.45 | 0.14± 0.03 | 0.37± 0.05 | 0.42± 0.12 | 0.33± 0.12 | 0.19± 0.07 | 0.22± 0.04 | 0.19± 0.02 | 0.15± 0.04 | 0.47± 0.17 |
| Phenolic Acids | Ferulic acid | 2.07± 0.66 | 0.53± 0.17 | 0.42± 0.09 | 1.48± 0.52 | 0.56± 0.34 | LOQ | LOQ | 0.18± 0.01 | 0.14± 0.03 | LOD |
| Phenolic Acids | p-Coumaric acid | 3.42± 1.00 | 1.25± 0.21 | 1.43± 0.11 | 1.30± 0.48 | 1.26± 0.59 | 0.61± 0.16 | 0.50± 0.18 | 0.79± 0.19 | 0.34± 0.08 | 0.28± 0.15 |
| Phenolic Acids | Quinic acid | 25.64± 4.74 | 23.85± 0.91 | 23.93± 1.32 | 18.27± 6.86 | 12.54± 1.79 | 16.11± 0.87 | 15.13± 0.58 | 14.34± 0.72 | 13.24± 1.08 | 10.75± 4.61 |
| Phenolic Acids | Sinapic acid | 0.39± 0.04 | 0.13± 0.05 | 0.09± 0.03 | 0.87± 0.07 | 0.17± 0.16 | LOD | LOD | LOD | LOD | LOD |
| Phenolic Acids | Syringic aldehyde | 1.07± 0.12 | 0.32± 0.03 | 0.30± 0.05 | 0.31± 0.08 | 0.91± 0.68 | 0.22± 0.02 | 0.26± 0.07 | 0.25± 0.04 | 0.12± 0.00 | 0.37± 0.09 |
| Sunflower Oil |  |  |  |  |  |  |  |  |  |  |  |
| Flavones | Amentoflavone | LOQ | LOQ | LOQ | LOQ | LOD | LOD | LOD | LOD | LOD | LOD |
| Flavones | Herbacetin | 77.61± 27.74 | 18.44± 3.86 | 9.41± 3.69 | 6.65± 2.54 | 4.10± 1.16 | 4.12± 0.88 | 3.01± 0.94 | 3.32± 0.70 | 2.88± 1.23 | 1.42± 0.73 |
| Flavonoids | Apigenin | LOQ | LOQ | LOQ | LOQ | LOD | LOD | LOD | LOD | LOD | LOD |
| Flavonoids | Chalcone | LOQ | LOQ | LOQ | LOQ | LOD | LOD | LOD | LOD | LOD | LOD |
| Flavonoids | Eriodictyol | 0.99± 0.21 | LOQ | 0.35± 0.06 | LOQ | LOQ | LOQ | LOQ | LOD | LOQ | 0.20± 0.13 |
| Flavonoids | Hesperetin | 3.83± 1.24 | 0.43± 0.08 | 0.31± 0.08 | LOQ | 0.23± 0.06 | LOQ | 0.33± 0.09 | 0.40± 0.08 | LOQ | 0.81± 0.29 |
| Flavonoids | Isorhamnetin | 0.59± 0.16 | 0.27± 0.01 | 0.35± 0.01 | 0.19± 0.05 | 0.22± 0.05 | 0.29± 0.07 | LOQ | LOQ | LOQ | LOQ |
| Flavonoids | Isorhamnetin 3-rutinoside | 1.17± 0.23 | 0.29± 0.10 | 0.21± 0.08 | 0.26± 0.05 | 0.38± 0.08 | LOQ | 0.25± 0.04 | 0.32± 0.07 | LOQ | LOQ |
| Flavonoids | Isorhamnetin-3-O-glucoside | 1.43± 0.22 | 0.13± 0.01 | 0.36± 0.05 | 0.25± 0.07 | 0.34± 0.10 | 0.22± 0.02 | 0.30± 0.10 | 0.20± 0.05 | 0.35± 0.03 | 0.14± 0.02 |
| Flavonoids | Kaempferol | 1.35± 0.31 | 0.23± 0.01 | 0.35± 0.08 | LOQ | 0.44± 0.12 | 0.32± 0.03 | 0.36± 0.08 | 0.32± 0.08 | 0.27± 0.08 | LOQ |
| Flavonoids | Kaempferol-3-O-glucoside (Astragalin) | 1.78± 0.20 | 0.41± 0.02 | 0.34± 0.08 | LOQ | 0.41± 0.06 | 0.31± 0.11 | 0.28± 0.05 | 0.28± 0.02 | LOQ | LOQ |
| Flavonoids | Kaempferol-3-O-glucuronic acid | 0.65± 0.14 | 0.44± 0.12 | 0.48± 0.17 | 0.48± 0.14 | 0.26± 0.09 | 0.31± 0.08 | 0.18± 0.07 | 0.48± 0.12 | 0.37± 0.02 | 0.50± 0.21 |
| Flavonoids | Luteolin | 0.16± 0.04 | 0.06± 0.01 | 0.05± 0.01 | 0.06± 0.01 | LOD | LOD | LOD | LOD | LOD | LOD |
| Flavonoids | Luteolin-7-O-glucoside | 0.26± 0.09 | LOD | LOD | LOD | LOD | LOD | LOD | LOD | LOD | LOD |
| Flavonoids | Naringenin | LOQ | LOQ | LOQ | LOQ | LOQ | LOQ | LOQ | LOQ | LOQ | LOQ |
| Flavonoids | Quercetin | 1.04± 0.28 | 0.15± 0.02 | 0.23± 0.03 | 0.18± 0.04 | 0.33± 0.04 | 0.42± 0.05 | LOQ | LOQ | LOD | LOQ |
| Flavonoids | Quercetin-3-O-glucoronic acid | 0.79± 0.25 | 0.13± 0.01 | 0.16± 0.02 | 0.18± 0.05 | 0.17± 0.04 | 0.17± 0.02 | 0.33± 0.01 | 0.24± 0.03 | 0.32± 0.08 | 0.27± 0.03 |
| Flavonoids | Quercetin-3-O-glucoside | 1.39± 0.15 | 0.22± 0.02 | 0.31± 0.02 | 0.30± 0.06 | 0.19± 0.05 | 0.27± 0.11 | 0.37± 0.12 | 0.14± 0.00 | 0.24± 0.02 | 0.13± 0.05 |
| Flavones | Amentoflavone | LOQ | LOQ | LOQ | LOQ | LOQ | LOD | LOD | LOD | LOD | LOD |
| Flavones | Herbacetin | 77.61± 27.74 | 18.44± 3.86 | 9.41± 3.69 | 6.65± 2.54 | 4.10± 1.16 | 4.12± 0.88 | 3.01± 0.94 | 3.32± 0.70 | 2.88± 1.23 | 1.42± 0.73 |
| Flavonoids | Apigenin | 0.18± 0.05 | LOQ | LOQ | LOQ | LOD | LOD | LOD | LOD | LOD | LOD |
| Flavonoids | Chalcone | 0.11± 0.03 | LOD | LOD | LOD | LOD | LOD | LOD | LOD | LOD | LOD |
| Lignans | Matairesinol | 0.75±0.18 | 0.59±0.33 | LOD | 0.18±0.07 | 0.34±0.15 | 0.29±0.08 | 0.47±0.22 | 0.33±0.18 | LOQ | LOD |
| Lignans | Pinoresinol | 0.62±0.16 | 0.13±0.05 | LOD | LOQ | LOD | LOD | LOD | LOD | LOD | LOD |
| Lignans | Secoisolariciresinol | 0.44±0.16 | LOQ | LOQ | LOQ | LOQ | LOQ | LOD | LOD | LOD | LOD |
| Phenolic Acids | 1,3-Dicaffeoylquinic acid | 0.29±0.03 | LOQ | LOQ | LOQ | LOQ | LOQ | LOD | LOD | LOD | LOD |
| Phenolic Acids | 3,4-Dimethoxycinnamic acid | 0.11±0.02 | 0.23±0.03 | 0.31±0.04 | 0.25±0.03 | 0.86±0.04 | 0.76±0.05 | 0.56±0.05 | 0.59±0.07 | 0.21±0.02 | LOD |
| Phenolic Acids | 4-Hydroxybenzoic acid | 0.54±0.12 | 0.31±0.11 | 0.27±0.01 | 0.26±0.02 | 0.24±0.07 | 0.23±0.09 | 0.33±0.07 | 0.29±0.02 | 0.39±0.09 | 0.26±0.03 |
| Phenolic Acids | 4-O-Caffeoylquinic acid | 0.04±0.00 | 0.02±0.00 | 0.03±0.00 | LOD | LOD | LOD | LOD | LOD | LOD | LOD |
| Phenolic Acids | Caffeic acid | 1.05±0.21 | 0.17±0.05 | 0.12±0.04 | 0.13±0.02 | 0.18±0.05 | LOQ | LOQ | LOQ | LOD | LOD |
| Phenolic Acids | Caffeic acid hexoside | 2.60±0.86 | 0.58±0.16 | 0.41±0.16 | 0.19±0.08 | 0.42±0.09 | 0.01±0.00 | 0.42±0.13 | 0.39±0.11 | 0.18±0.09 | 0.13±0.07 |
| Phenolic Acids | Chlorogenic acid | 2.92±1.14 | 0.26±0.15 | 0.23±0.13 | LOQ | LOQ | LOQ | LOQ | LOQ | LOD | LOD |
| Phenolic Acids | cis Cinnamic acid | 2.15±0.76 | 0.20±0.08 | 0.21±0.13 | 0.11±0.05 | 0.17±0.04 | 0.11±0.05 | LOQ | LOQ | LOQ | LOQ |
| Phenolic Acids | Ellagic acid | 16.65±6.51 | 3.13±0.44 | 1.69±0.73 | 0.69±0.29 | 0.94±0.17 | 0.71±0.34 | 0.30±0.09 | 0.55±0.08 | 0.49±0.10 | 0.45±0.28 |
| Phenolic Acids | Ferulic acid | 0.80±0.06 | 0.12±0.02 | 0.13±0.02 | 0.14±0.03 | LOQ | LOQ | LOQ | LOQ | LOQ | LOD |
| Phenolic Acids | p-Coumaric acid | 7.50±2.17 | 1.35±0.37 | 1.48±0.54 | 1.17±0.24 | 0.71±0.20 | 0.72±0.14 | 0.51±0.21 | 1.46±0.41 | 0.48±0.12 | 0.36±0.25 |
| Phenolic Acids | Quinic acid | 20.63±1.98 | 23.88±1.07 | 23.15±1.20 | 32.03±1.96 | 15.30±0.86 | 13.59±0.77 | 12.59±0.79 | 10.51±0.47 | 9.47±0.42 | 4.54±3.11 |
| HO Sunflower Oil |  |  |  |  |  |  |  |  |  |  |  |
| Flavones | Herbacetin | 39.76± 14.50 | 3.92± 0.88 | 7.56± 2.77 | 1.32± 0.29 | 2.59± 0.70 | 2.62± 0.75 | 2.60± 0.92 | 2.22± 1.15 | 1.83± 0.36 | 1.36± 0.78 |
| Flavonoids | Apigenin | LOQ | LOQ | LOQ | LOQ | LOQ | LOQ | LOD | LOD | LOD | LOD |
| Flavonoids | Chalcone | LOD | LOD | LOD | LOD | LOD | LOD | LOD | LOD | LOD | LOD |
| Flavonoids | Eriodictyol | 0.69± 0.15 | 0.20± 0.04 | 0.35± 0.09 | 0.18± 0.01 | 0.20± 0.07 | LOQ | LOD | 0.49± 0.08 | LOQ | LOQ |
| Flavonoids | Hesperetin | 2.35± 0.62 | 0.32± 0.13 | 0.37± 0.12 | LOQ | LOQ | LOQ | LOQ | 0.38± 0.21 | LOQ | LOD |
| Flavonoids | Hesperidin | 0.33± 0.03 | LOQ | LOQ | LOQ | LOQ | LOQ | LOQ | LOD | LOD | LOD |
| Flavonoids | Isorhamnetin 3-rutinoside | 0.79± 0.31 | 0.25± 0.02 | 0.38± 0.14 | 0.38± 0.03 | LOQ | LOQ | LOQ | LOQ | LOQ | LOQ |
| Flavonoids | Isorhamnetin-3-O-glucoside | 0.39± 0.18 | 0.36± 0.03 | 0.21± 0.02 | 0.37± 0.08 | LOQ | 0.36± 0.02 | LOQ | LOQ | LOQ | LOQ |
| Flavonoids | Kaempferol | 1.15± 0.29 | LOQ | 0.46± 0.05 | 0.42± 0.04 | 0.29± 0.04 | LOQ | 0.30± 0.02 | 0.45± 0.16 | 0.48± 0.03 | LOQ |
| Flavonoids | Kaempferol-3-O-glucoside (Astragalin) | LOQ | LOD | LOQ | LOQ | LOQ | LOQ | LOQ | 0.82± 0.42 | 0.29± 0.09 | LOD |
| Flavonoids | Kaempferol-3-O-glucuronic acid | LOQ | LOQ | 0.38± 0.02 | LOQ | 0.37± 0.04 | LOQ | 0.44± 0.08 | 0.64± 0.33 | LOQ | 0.32± 0.10 |
| Flavonoids | Luteolin | LOQ | LOD | LOD | LOD | LOD | LOD | LOD | LOD | LOD | LOD |
| Flavonoids | Quercetin | 0.56± 0.10 | LOD | LOD | LOD | LOD | LOD | LOD | LOD | LOD | LOD |
| Flavonoids | Quercetin-3-O-glucoside | 0.75± 0.05 | LOQ | 0.27± 0.00 | 0.23± 0.07 | 0.38± 0.08 | LOQ | 0.30± 0.07 | 0.17± 0.07 | 0.26± 0.01 | 0.15± 0.04 |
| Lignans | Matairesinol | 0.22± 0.07 | LOQ | LOQ | LOQ | LOQ | LOD | LOQ | LOQ | LOD | LOD |
| Lignans | Pinoresinol | LOQ | LOQ | LOQ | LOQ | LOQ | LOQ | LOQ | 0.24± 0.14 | LOD | LOD |
| Lignans | Secoisolariciresinol | LOQ | LOD | LOD | LOD | LOD | LOD | LOD | LOD | LOD | LOD |
| Phenolic Acids | 1,3-Dicaffeoylquinic acid | 0.20±0.03 | 0.30±0.04 | LOQ | LOQ | LOQ | LOD | LOD | LOD | LOD | LOD |
| Phenolic Acids | 3,4-Dimethoxycinnamic acid | 0.41±0.10 | 0.31±0.02 | 0.29±0.03 | 0.42±0.07 | 0.86±0.05 | 0.76±0.07 | 0.61±0.04 | 0.65±0.02 | 0.40±0.07 | LOD |
| Phenolic Acids | 4-Hydroxybenzoic acid | LOQ | LOQ | 0.20±0.03 | 0.17±0.04 | 0.36±0.08 | 0.31±0.11 | 0.17±0.05 | 0.25±0.08 | 0.31±0.11 | 0.29±0.11 |
| Phenolic Acids | 4-O-Caffeoylquinic acid | 0.51±0.14 | 0.13±0.06 | LOQ | LOD | LOD | LOD | LOD | LOD | LOD | LOD |
| Phenolic Acids | Caffeic acid | 0.25±0.08 | 0.12±0.06 | LOQ | LOQ | 0.11±0.01 | LOQ | LOD | 0.10±0.03 | LOD | LOD |
| Phenolic Acids | Caffeic acid hexoside | 4.62±2.26 | 0.85±0.21 | LOQ | LOQ | LOQ | LOQ | LOQ | LOQ | LOD | LOD |
| Phenolic Acids | Chlorogenic acid | 0.54±0.09 | LOQ | 0.76±0.19 | LOQ | LOQ | LOQ | LOQ | LOQ | LOD | LOD |
| Phenolic Acids | cis Cinnamic acid | 10.39±3.37 | 2.44±1.19 | LOQ | LOQ | LOQ | LOQ | LOQ | LOQ | LOD | LOD |
| Phenolic Acids | Ellagic acid | 1.51±0.69 | 0.20±0.08 | 1.77±0.66 | 0.26±0.01 | 0.37±0.06 | LOQ | 0.29±0.09 | 1.06±0.70 | 0.24±0.03 | 0.25±0.14 |
| Phenolic Acids | Ferulic acid | 2.81±0.69 | 0.90±0.31 | LOQ | LOQ | LOQ | LOQ | LOQ | LOD | LOD | LOD |
| Phenolic Acids | p-Coumaric acid | 0.67±0.18 | 0.04±0.01 | 1.19±0.27 | 0.83±0.10 | 0.38±0.18 | 0.56±0.12 | 0.57±0.14 | 0.33±0.27 | 0.38±0.10 | 0.33±0.20 |
| Phenolic Acids | Protocatechuic acid | 23.17±1.43 | 23.73±3.72 | 24.45±1.44 | 30.93±2.76 | 15.59±0.68 | 14.14±1.23 | 15.29±1.02 | 14.69±1.01 | 11.31±1.03 | 7.48±5.38 |
| Phenolic Acids | Quinic acid | 0.20±0.03 | 0.30±0.04 | LOD | LOD | LOD | LOD | LOD | LOD | LOD | LOD |
| Linseed Oil |  |  |  |  |  |  |  |  |  |  |  |
| Flavan-3-Ol | Gallocatechol | 0.40± 0.11 | LOQ | LOQ | LOQ | LOQ | LOQ | LOQ | LOQ | LOD | LOD |
| Flavones | Amentoflavone | LOQ | LOD | LOD | LOD | LOD | LOD | LOD | LOD | LOD | LOD |
| Flavones | Herbacetin | 36.21± 4.84 | 11.70± 2.15 | 5.39± 0.82 | 1.21± 0.22 | 2.40± 0.36 | 1.81± 0.26 | 1.99± 0.26 | 1.75± 0.33 | 1.67± 0.31 | 1.29± 0.37 |
| Flavonoids | Apigenin | LOQ | LOD | LOD | LOD | LOD | LOD | LOD | LOD | LOD | LOD |
| Flavonoids | Chalcone | LOQ | LOD | LOD | LOD | LOD | LOD | LOD | LOD | LOD | LOD |
| Flavonoids | Eriodictyol | 0.34± 0.07 | LOQ | 0.35± 0.07 | LOQ | 0.19± 0.07 | LOQ | LOQ | 0.49± 0.08 | LOQ | LOQ |
| Flavonoids | Hesperetin | 3.22± 1.22 | 1.36± 0.25 | 0.33± 0.08 | LOQ | LOQ | LOQ | LOD | LOQ | LOD | LOQ |
| Flavonoids | Isorhamnetin 3-rutinoside | 0.55± 0.19 | 0.21± 0.08 | LOQ | 0.30± 0.04 | LOQ | 0.37± 0.12 | 0.31± 0.06 | LOQ | LOQ | LOQ |
| Flavonoids | Kaempferol | 0.52± 0.07 | 0.39± 0.14 | 0.46± 0.17 | 0.40± 0.00 | 0.35± 0.05 | 0.31± 0.04 | 0.34± 0.02 | LOQ | LOQ | LOQ |
| Flavonoids | Luteolin | 0.25± 0.09 | LOD | LOD | LOD | LOD | LOD | LOD | LOD | LOD | LOD |
| Flavonoids | Luteolin-7-O-glucoside | 0.17± 0.05 | LOD | LOD | LOD | LOD | LOD | LOD | LOD | LOD | LOD |
| Flavonoids | Naringin | 0.20± 0.09 | LOQ | LOQ | LOQ | LOQ | LOQ | LOD | LOQ | LOQ | LOQ |
| Flavonoids | Quercetin-3-O-glucoronic acid | 0.40± 0.11 | 0.27± 0.08 | LOQ | 0.35± 0.09 | LOQ | 0.28± 0.09 | 0.26± 0.00 | 0.37± 0.13 | LOQ | LOQ |
| Lignans | Matairesinol | 0.29± 0.09 | 0.47± 0.12 | 0.46± 0.10 | LOQ | 0.24± 0.07 | 0.31± 0.12 | 0.52± 0.24 | 0.62± 0.37 | LOQ | LOQ |
| Lignans | Pinoresinol | 0.28± 0.07 | LOQ | LOQ | LOD | LOD | LOD | LOD | LOD | LOD | LOD |
| Lignans | Secoisolariciresinol | 5.24± 1.88 | 1.44± 0.24 | 0.45± 0.07 | 0.11± 0.02 | LOD | LOD | LOD | LOD | LOD | LOD |
| Phenolic Acids | 1,3-Dicaffeoylquinic acid | LOQ | LOD | LOD | LOD | LOD | LOD | LOD | LOD | LOD | LOD |
| Phenolic Acids | 3,4-Dimethoxycinnamic acid | LOQ | 0.22± 0.02 | LOQ | 0.34± 0.08 | 0.49± 0.04 | 0.53± 0.08 | 0.44± 0.06 | 0.34± 0.03 | LOQ | LOD |
| Phenolic Acids | 4-Hydroxybenzoic acid | 0.95± 0.30 | 0.35± 0.05 | LOQ | 0.31± 0.07 | LOQ | LOQ | 0.37± 0.07 | LOQ | 0.38± 0.12 | 0.41± 0.06 |
| Phenolic Acids | Caffeic acid | 0.36± 0.07 | LOQ | 0.38± 0.12 | 0.35± 0.12 | 0.60± 0.09 | 0.39± 0.08 | 0.38± 0.02 | 0.49± 0.09 | 0.36± 0.10 | LOQ |
| Phenolic Acids | Caffeic acid hexoside | 0.34± 0.07 | LOQ | LOQ | LOQ | LOQ | LOQ | LOQ | LOQ | LOD | LOD |
| Phenolic Acids | Chlorogenic acid | 0.37± 0.07 | LOQ | LOQ | LOQ | LOQ | LOQ | LOQ | LOQ | LOD | LOD |
| Phenolic Acids | cis Cinnamic acid | 0.71± 0.24 | LOQ | LOQ | LOQ | LOD | LOD | LOD | LOD | LOD | LOD |
| Phenolic Acids | Coumaric acid hexoside | 0.11± 0.01 | 0.03± 0.01 | LOD | LOD | LOD | LOD | LOD | LOD | LOD | LOD |
| Phenolic Acids | Ellagic acid | 1.07± 0.25 | LOQ | LOQ | 0.32± 0.08 | LOQ | LOQ | LOQ | LOD | 0.32± 0.01 | LOQ |
| Phenolic Acids | Ferulic acid | 1.24± 0.26 | 0.23± 0.07 | LOQ | LOQ | LOQ | LOQ | LOQ | LOQ | LOQ | LOD |
| Phenolic Acids | p-Coumaric acid | 6.07± 2.55 | 1.09± 0.23 | 1.57± 0.18 | 0.82± 0.11 | 0.44± 0.15 | 0.51± 0.11 | 0.48± 0.11 | 0.58± 0.18 | 0.41± 0.07 | LOQ |
| Phenolic Acids | Quinic acid | 22.74± 1.86 | 26.48± 1.35 | 23.36± 1.39 | 22.92± 1.31 | 15.37± 1.17 | 13.43± 1.17 | 15.40± 0.80 | 15.15± 1.04 | 12.28± 0.76 | 5.23± 3.01 |
| Hempseed Oil |  |  |  |  |  |  |  |  |  |  |  |
| Flavan-3-Ol | Epicatechin | 3.0 ± 1.2 | 1.9 ± 0.6 | 1.78 ± 0.46 | 0.74 ± 0.29 | 1.95 ± 0.17 | 0.99 ± 0.14 | 0.71 ± 0.28 | 0.94 ± 0.03 | 1.88 ± 0.02 | 2.34 ± 0.63 |
| Flavan-3-Ol | Gallocatechol | 1.3 ± 0.3 | 0.3 ± 0.1 | LOQ | LOQ | LOQ | LOQ | LOD | LOQ | LOD | LOD |
| Flavones | Amentoflavone | 0.3 ± 0.1 | LOD | LOD | LOD | LOD | LOD | LOD | LOD | LOD | LOD |
| Flavones | Herbacetin | 19.0 ± 4.3 | 13.6 ± 2.9 | 1.72 ± 0.44 | 0.19 ± 0.04 | 0.41 ± 0.11 | 0.4 ± 0.06 | 0.48 ± 0.17 | 0.35 ± 0.01 | 0.37 ± 0.13 | 0.25 ± 0.07 |
| Flavonoids | Apigenin | 0.1 ± 0.0 | 0.0 ± 0.0 | LOD | LOD | LOD | LOD | LOD | LOD | LOD | LOD |
| Flavonoids | Chalcone | 0.1 ± 0.0 | 0.1 ± 0.0 | 0.14 ± 0.03 | LOD | LOD | LOD | LOD | LOD | LOD | LOD |
| Flavonoids | Eriodictyol | 0.3 ± 0.1 | LOQ | 0.37 ± 0.09 | LOQ | LOQ | LOQ | LOQ | LOD | LOD | LOD |
| Flavonoids | Hesperetin | 7.1 ± 1.7 | 2.2 ± 0.9 | 0.98 ± 0.47 | 0.75 ± 0.43 | 0.85 ± 0.39 | LOQ | LOQ | LOQ | LOQ | LOD |
| Flavonoids | Hesperidin | 2.0 ± 0.8 | 0.3 ± 0.1 | 0.42 ± 0.08 | LOQ | LOQ | LOQ | LOQ | LOD | LOD | LOQ |
| Flavonoids | Isorhamnetin 3-rutinoside | 4.1 ± 0.8 | 0.5 ± 0.1 | LOQ | LOQ | 2.4 ± 1.25 | LOQ | 0.48 ± 0.22 | 0.36 ± 0.07 | 0.27 ± 0.09 | 0.19 ± 0.14 |
| Flavonoids | Isorhamnetin-3-O-glucoside | 1.0 ± 0.4 | LOQ | LOQ | LOQ | LOQ | LOQ | LOQ | LOQ | LOQ | LOD |
| Flavonoids | Kaempferol | 1.4 ± 0.2 | 0.4 ± 0.1 | 0.42 ± 0.09 | 0.59 ± 0.24 | 0.78 ± 0.35 | LOQ | LOQ | LOQ | LOQ | LOQ |
| Flavonoids | Kaempferol-3-O-arabinoside | 4.9 ± 0.3 | 1.8 ± 0.5 | LOQ | LOQ | LOQ | LOQ | LOQ | 0.44 ± 0.04 | LOQ | LOQ |
| Flavonoids | Kaempferol-3-O-glucoside (Astragalin) | 0.7 ± 0.2 | 0.3 ± 0.0 | LOQ | 0.29 ± 0.0 | 0.49 ± 0.06 | 0.42 ± 0.15 | 0.34 ± 0.07 | 0.36 ± 0.13 | LOQ | LOQ |
| Flavonoids | Kaempferol-3-O-glucuronic acid | 0.5 ± 0.2 | 0.2 ± 0.0 | LOQ | LOQ | LOQ | LOQ | LOD | LOQ | LOD | LOQ |
| Flavonoids | Luteolin | LOQ | LOD | LOD | LOD | LOD | LOD | LOD | LOD | LOD | LOD |
| Flavonoids | Luteolin-7-O-glucoside | 0.3 ± 0.2 | LOQ | LOD | LOD | LOD | LOD | LOD | LOD | LOD | LOD |
| Flavonoids | Naringenin | 0.2 ± 0.0 | LOQ | LOQ | LOQ | LOQ | LOQ | LOQ | LOQ | LOQ | LOQ |
| Flavonoids | Naringin | 1.7 ± 0.0 | LOQ | LOQ | LOQ | LOQ | LOQ | LOQ | LOQ | LOQ | LOD |
| Flavonoids | Quercetin | LOQ | LOQ | LOQ | LOQ | LOQ | LOQ | LOQ | LOQ | LOD | LOD |
| Flavonoids | Quercetin-3-O-glucoronic acid | 0.8 ± 0.3 | 0.3 ± 0.1 | LOQ | 0.25 ± 0.03 | 0.36 ± 0.13 | LOQ | LOQ | 0.41 ± 0.11 | 0.23 ± 0.08 | 0.29 ± 0.12 |
| Lignans | Lariciresinol | 0.1 ± 0.0 | 0.0 ± 0.0 | 0.07 ± 0.0 | LOD | LOD | LOD | LOD | LOD | LOD | LOD |
| Lignans | Matairesinol | 2.1 ± 0.2 | 1.3 ± 0.4 | 1.13 ± 0.39 | 1.16 ± 0.43 | 3.11 ± 0.65 | 0.54 ± 0.17 | 2.24 ± 1.01 | 0.73 ± 0.22 | LOQ | LOD |
| Lignans | Pinoresinol | 1.6 ± 0.1 | 0.6 ± 0.2 | 0.4 ± 0.15 | 0.7 ± 0.32 | LOQ | LOQ | LOQ | LOQ | LOD | LOD |
| Lignans | Secoisolariciresinol | 5.5 ± 2.6 | 1.1 ± 0.4 | 0.38 ± 0.19 | LOQ | LOQ | LOQ | LOD | LOD | LOQ | LOQ |
| Phenolic Acids | 3,4-Dimethoxycinnamic acid | LOQ | LOQ | 0.27 ± 0.08 | 0.36 ± 0.1 | 0.82 ± 0.13 | 0.84 ± 0.05 | 0.68 ± 0.06 | 0.65 ± 0.06 | LOD | LOD |
| Phenolic Acids | 4-O-Caffeoylquinic acid | 0.2 ± 0.1 | LOD | LOD | LOD | LOD | LOD | LOD | LOD | LOD | LOD |
| Phenolic Acids | 4-Hydroxybenzoic acid | 2.0 ± 0.4 | 0.3 ± 0.1 | LOQ | 0.98 ± 0.51 | 1.36 ± 0.86 | 0.31 ± 0.06 | LOQ | 0.36 ± 0.05 | LOQ | LOQ |
| Phenolic Acids | Caffeic acid | LOQ | LOQ | LOQ | LOQ | LOD | LOD | LOD | LOD | 0.19 ± 0.1 | LOD |
| Phenolic Acids | Caffeic acid hexoside | 1.6 ± 0.3 | 0.6 ± 0.2 | 0.48 ± 0.15 | 0.6 ± 0.28 | 1.07 ± 0.46 | LOQ | 0.46 ± 0.2 | 0.38 ± 0.13 | LOQ | LOD |
| Phenolic Acids | Chlorogenic acid | 3.5 ± 2.4 | 0.7 ± 0.3 | 0.46 ± 0.19 | LOQ | LOQ | LOQ | 0.44 ± 0.08 | LOQ | LOD | LOD |
| Phenolic Acids | Coumaric acid hexoside | 0.1 ± 0.0 | LOD | LOD | LOD | LOD | LOD | LOD | LOD | LOD | LOD |
| Phenolic Acids | Ellagic acid | 2.4 ± 1.1 | 0.7 ± 0.1 | 0.17 ± 0.02 | 0.75 ± 0.24 | 0.46 ± 0.23 | LOQ | 0.18 ± 0.01 | 0.34 ± 0.09 | 1.52 ± 0.32 | 0.23 ± 0.05 |
| Phenolic Acids | Ferulic acid | 1.1 ± 0.2 | LOQ | LOQ | LOQ | LOQ | LOQ | LOD | LOD | LOD | LOD |
| Phenolic Acids | Gallic acid | 0.6 ± 0.2 | LOQ | LOQ | LOQ | LOQ | LOQ | LOQ | LOQ | 0.92 ± 0.43 | 0.82 ± 0.25 |
| Phenolic Acids | Quinic acid | 25.3 ± 1.1 | 22.1 ± 1.5 | 20.93 ± 1.12 | 21.15 ± 5.01 | 12.51 ± 1.38 | 12.0 ± 0.83 | 12.66 ± 1.39 | 12.21 ± 1.23 | 8.91 ± 2.36 | 4.23 ± 3.08 |
| Phenolic Acids | cis Cinnamic acid | 1.3 ± 0.3 | LOQ | LOQ | LOQ | LOQ | LOD | LOD | LOD | LOD | LOD |
| Phenolic Acids | p-Coumaric acid | 7.2 ± 2.7 | 1.8 ± 0.8 | 4.03 ± 1.7 | 2.84 ± 0.98 | 1.37 ± 0.72 | 0.8 ± 0.23 | 0.85 ± 0.21 | 1.25 ± 0.47 | 0.91 ± 0.04 | 0.39 ± 0.32 |

*<LOD: below limit of detection (determined with S/N<3), <LOQ: below limit of quantification (S/N<10)*

***Table S7:*** *Quantitative results of the GC-MS measurements. Data given as mean* ± *standard deviation (SD) in ppm, n=9.*

| Oil Type | 1-Hexanol [ppm] | Hexanal [ppm] | 2-Heptanone [ppm] | 2-Pentanone [ppm] |  |
| --- | --- | --- | --- | --- | --- |
| Black cumin Oil |  |  |  |  |  |
| Day 0 | 0.00 ± 0.00 | 0.02 ± 0.00 |  |  |  |
| 3 | 0.01 ± 0.00 | 0.08 ± 0.01 |  |  |  |
| 7 | 0.01 ± 0.00 | 0.12 ± 0.02 |  |  |  |
| 14 | 0.02 ± 0.01 | 0.28 ± 0.11 |  |  |  |
| 28 | 0.06 ± 0.01 | 0.33 ± 0.12 |  |  |  |
| 42 | 0.06 ± 0.02 | 0.66 ± 0.11 |  |  |  |
| 56 | 0.06 ± 0.01 | 0.63 ± 0.06 |  |  |  |
| 84 | 0.15 ± 0.04 | 1.37 ± 0.30 |  |  |  |
| 112 | 0.17 ± 0.03 | 1.45 ± 0.21 |  |  |  |
| 168 | 0.06 ± 0.01 | 1.17 ± 0.14 |  |  |  |
| Canola Oil |  |  |  |  |  |
| Day 0 | 0.00 ± 0.00 | 0.02 ± 0.01 |  |  |  |
| 3 | 0.01 ± 0.00 | 0.06 ± 0.01 |  |  |  |
| 7 | 0.01 ± 0.00 | 0.10 ± 0.02 |  |  |  |
| 14 | 0.02 ± 0.01 | 0.31 ± 0.06 |  |  |  |
| 28 | 0.06 ± 0.02 | 0.45 ± 0.11 |  |  |  |
| 42 | 0.06 ± 0.00 | 0.65 ± 0.19 |  |  |  |
| 56 | 0.04 ± 0.02 | 0.58 ± 0.04 |  |  |  |
| 84 | 0.08 ± 0.00 | 0.76 ± 0.17 |  |  |  |
| 112 | 0.10 ± 0.02 | 0.84 ± 0.25 |  |  |  |
| 168 | 0.08 ± 0.02 | 0.96 ± 0.18 |  |  |  |
| Sunflower Oil |  |  |  |  |  |
| Day 0 | 0.02 ± 0.01 | 0.02 ± 0.00 |  |  |  |
| 3 | 0.01 ± 0.01 | 0.04 ± 0.01 |  |  |  |
| 7 | 0.01 ± 0.01 | 0.06 ± 0.01 |  |  |  |
| 14 | 0.02 ± 0.00 | 0.47 ± 0.17 |  |  |  |
| 28 | 0.06 ± 0.03 | 0.69 ± 0.21 |  |  |  |
| 42 | 0.10 ± 0.02 | 0.85 ± 0.38 |  |  |  |
| 56 | 0.15 ± 0.02 | 1.24 ± 0.29 |  |  |  |
| 84 | 0.11 ± 0.02 | 1.61 ± 0.37 |  |  |  |
| 112 | 0.22 ± 0.05 | 1.81 ± 0.51 |  |  |  |
| 168 | 0.13 ± 0.01 | 2.13 ± 0.12 |  |  |  |
| HO Sunflower Oil |  |  |  |  |  |
| Day 0 | 0.02 ± 0.01 | 0.02 ± 0.00 |  |  |  |
| 3 | 0.02 ± 0.01 | 0.04 ± 0.01 |  |  |  |
| 7 | 0.01 ± 0.00 | 0.06 ± 0.01 |  |  |  |
| 14 | 0.02 ± 0.00 | 0.47 ± 0.07 |  |  |  |
| 28 | 0.06 ± 0.01 | 0.83 ± 0.13 |  |  |  |
| 42 | 0.10 ± 0.03 | 0.97 ± 0.26 |  |  |  |
| 56 | 0.15 ± 0.03 | 1.55 ± 0.33 |  |  |  |
| 84 | 0.11 ± 0.02 | 3.87 ± 0.89 |  |  |  |
| 112 | 0.22 ± 0.08 | 2.17 ± 1.09 |  |  |  |
| 168 | 0.13 ± 0.03 | 1.28 ± 0.58 |  |  |  |
| Linseed Oil |  |  |  |  |  |
| Day 0 | 0.04 ± 0.02 | 0.03 ± 0.01 | 0.00 ± 0.00 |  |  |
| 3 | 0.05 ± 0.02 | 0.05 ± 0.02 | 0.01 ± 0.00 |  |  |
| 7 | 0.06 ± 0.01 | 0.08 ± 0.03 | 0.02 ± 0.01 |  |  |
| 14 | 0.12 ± 0.03 | 0.71 ± 0.27 | 0.02 ± 0.01 |  |  |
| 28 | 0.30 ± 0.09 | 5.08 ± 1.68 | 0.03 ± 0.01 |  |  |
| 42 | 0.46 ± 0.12 | 18.32 ± 6.83 | 0.06 ± 0.02 |  |  |
| 56 | 0.64 ± 0.22 | 10.51 ± 2.61 | 0.04 ± 0.02 |  |  |
| 84 | 0.58 ± 0.07 | 18.50 ± 3.08 | 0.03 ± 0.01 |  |  |
| 112 | 0.60 ± 0.27 | 13.33 ± 2.57 | 0.05 ± 0.01 |  |  |
| 168 | 0.37 ± 0.03 | 5.72 ± 1.18 | 0.02 ± 0.00 |  |  |
| Hempseed Oil |  |  |  |  |  |
| Day 0 | 0.07 ± 0.01 | 0.07 ± 0.01 | 0.01 ± 0.00 | 0.01 ± 0.00 |  |
| 3 | 0.09 ± 0.01 | 0.10 ± 0.02 | 0.02 ± 0.00 | 0.01 ± 0.00 |  |
| 7 | 0.09 ± 0.00 | 0.12 ± 0.03 | 0.02 ± 0.01 | 0.01 ± 0.00 |  |
| 14 | 0.18 ± 0.03 | 0.66 ± 0.20 | 0.02 ± 0.00 | 0.01 ± 0.00 |  |
| 28 | 0.36 ± 0.07 | 6.73 ± 1.66 | 0.03 ± 0.00 | 0.02 ± 0.00 |  |
| 42 | 0.51 ± 0.05 | 28.87 ± 2.86 | 0.07 ± 0.01 | 0.02 ± 0.00 |  |
| 56 | 0.78 ± 0.16 | 24.38 ± 0.54 | 0.05 ± 0.01 | 0.03 ± 0.00 |  |
| 84 | 0.81 ± 0.20 | 27.86 ± 3.61 | 0.06 ± 0.00 | 0.04 ± 0.00 |  |
| 112 | 0.76 ± 0.06 | 29.61 ± 7.38 | 0.08 ± 0.01 | 0.05 ± 0.01 |  |
| 168 | 0.73 ± 0.01 | 25.77 ± 8.12 | 0.11 ± 0.01 | 0.06 ± 0.01 |  |

***Table S8:*** *Results of volatiles analyzed via GC-MS. Data are given as mean* ± *standard deviation (SD) in area under the curve (AuC), n=9.*

| Oil Type | Day 0 | Day 3 | Day 7 | Day 14 | Day 28 | Day 42 | Day 56 | Day 84 | Day 112 | Day 168 |
| --- | --- | --- | --- | --- | --- | --- | --- | --- | --- | --- |
| Black cumin Oil |  |  |  |  |  |  |  |  |  |  |
| (E)-2-Octenal | 1814032 ± 238846 | 1819626 ± 351657 | 3406733 ± 34603 | 376867 ± 33912 | 308100 ± 56062 | 610851 ± 115310 | 985905 ± 843218 | 1858718 ± 140982 | 3283767 ± 194190 | 6286856 ± 249311 |
| 1-Octen-3-ol | 1744145 ± 542885 | 1261519 ± 318186 | 207415 ± 21670 | 21198 ± 10710 | 20002 ± 4552 | 140156 ± 59396 | 71717 ± 17997 | 174247 ± 47269 | 353721 ± 70634 | 81831 ± 16019 |
| 2-Decen-1-ol | 1467131 ± 205479 | 2492981 ± 127244 | 110971 ± 1521 | 71377 ± 7421 | 98568 ± 9616 | 133927 ± 18467 | 224148 ± 14972 | 51126 ± 14503 | 56216 ± 18655 | 76477 ± 5706 |
| 2-Octen-1-ol | 324867 ± 90511 | 233405 ± 174651 | 15085 ± 5162 | 10438 ± 3011 | 19677 ± 2017 | 33918 ± 6143 | 39828 ± 8209 | 83266 ± 2794 | 103792 ± 13487 | 103937 ± 26483 |
| 6-Oxabicyclo [3.1.0]hexan-3-ol | 176555 ± 12098 | 150664 ± 7361 | 90996 ± 21127 | 137189 ± 11021 | 168276 ± 44423 | 151689 ± 31013 | 188640 ± 25270 | 197573 ± 43705 | 236859 ± 4290 | 868122 ± 396428 |
| (E)-2-Decenal | 12698 ± 3669 | 13464 ± 4345 | 1914710 ± 414185 | 173154 ± 15780 | 145823 ± 25496 | 3398167 ± 530742 | 6554391 ± 404072 | 800696 ± 295421 | 1520851 ± 255892 | 869496 ± 152583 |
| (E)-2-Nonenal | 134810 ± 86201 | 185971 ± 54526 | 945660 ± 111820 | 1314165 ± 147815 | 1163775 ± 52574 | 5487132 ± 817286 | 6956830 ± 865767 | 5146693 ± 1047062 | 4321810 ± 949947 | 2539703 ± 518056 |
| 4,5-Epoxy-(E)-2-decenal | 319978 ± 25265 | 168363 ± 17967 | 189464 ± 35918 | 188188 ± 138084 | 132703 ± 21500 | 283722 ± 89344 | 440474 ± 11713 | 734743 ± 127673 | 940997 ± 42384 | 2007405 ± 907617 |
| Heptanal | 79854 ± 20965 | 81923 ± 23740 | 1366942 ± 90819 | 2018176 ± 354175 | 715476 ± 492624 | 4339881 ± 814078 | 4165367 ± 451962 | 4960221 ± 1514536 | 7584147 ± 1760053 | 6161253 ± 346332 |
| Nonanal | 822840 ± 28635 | 1233942 ± 68030 | 1778205 ± 341746 | 71781448 ± 39776234 | 274953 ± 102615 | 106930668 ± 50760127 | 195928107 ± 140972936 | 494157945 ± 84525833 | 12474410 ± 1146555 | 1877601 ± 1346242 |
| 5-Ethyl-2(5)-furanone | 43877740 ± 19955546 | 25886921 ± 10879349 | 46825 ± 13750 | 153904 ± 63349 | 72914 ± 19212 | 705269 ± 182465 | 508809 ± 46103 | 679853 ± 12182 | 1069533 ± 552839 | 513528 ± 81374 |
| Canola Oil |  |  |  |  |  |  |  |  |  |  |
| (E)-2-Decenal | 1080101 ± 96764 | 1139515 ± 207639 | 1039928 ± 347859 | 113827 ± 35086 | 158089 ± 58538 | 2463322 ± 349052 | 3009709 ± 429363 | 742837 ± 2553 | 809419 ± 376572 | 476198 ± 106725 |
| (E)-2-Nonenal | 444296 ± 77083 | 519612 ± 91645 | 580579 ± 96322 | 1200185 ± 259314 | 872553 ± 145985 | 2530206 ± 250338 | 1463128 ± 362301 | 2244442 ± 478786 | 2078247 ± 323404 | 1464372 ± 103559 |
| 2,4-Decadienal | 54698 ± 11141 | 84045 ± 10177 | 143056 ± 10240 | 76369 ± 13985 | 142923 ± 5714 | 233211 ± 58694 | 256621 ± 62592 | 618702 ± 183001 | 773629 ± 4537 | 772176 ± 23228 |
| 2,4-Heptadienal | 58649 ± 11155 | 136129 ± 9504 | 188185 ± 7929 | 208381 ± 23751 | 294508 ± 56076 | 328809 ± 41857 | 170354 ± 28311 | 304372 ± 75256 | 100409 ± 13480 | 64129 ± 9273 |
| Heptanal | 1174405 ± 217990 | 918220 ± 224253 | 946195 ± 286515 | 900617 ± 213011 | 880076 ± 199901 | 1505704 ± 320353 | 939369 ± 305482 | 1757077 ± 430067 | 4163854 ± 1106735 | 3298991 ± 977316 |
| Sunflower Oil |  |  |  |  |  |  |  |  |  |  |
| Heptanal | 1080101 ± 326490 | 1070614 ± 329099 | 946195 ± 355624 | 900617 ± 171960 | 880076 ± 168038 | 1505704 ± 489519 | 939369 ± 270417 | 1757077 ± 338874 | 4163854 ± 1747447 | 3298991 ± 1543662 |
| (E)-2-Decenal | 444296 ± 201411 | 547796 ± 208946 | 1039928 ± 339028 | 113827 ± 19458 | 158089 ± 27025 | 2463322 ± 499575 | 3009709 ± 259663 | 742837 ± 257779 | 809419 ± 127689 | 476198 ± 36950 |
| (E)-2-Nonenal | 54698 ± 29378 | 102931 ± 22965 | 580579 ± 286210 | 1200185 ± 246690. | 872553 ± 179348 | 2530206 ± 886492 | 1463128 ± 614595 | 2244442 ± 335940 | 2078247 ± 893332 | 1464372 ± 148958 |
| 2,4-Decadienal | 58649 ± 36185 | 131809 ± 34984 | 143056 ± 14742 | 76369 ± 14015 | 142923 ± 26229 | 233211 ± 54175 | 256621 ± 37289 | 618702 ± 35328 | 773629 ± 147835 | 772176 ± 114263 |
| 2,4-Heptadienal | 1174405 ± 497757 | 1124978 ± 479173 | 188185 ± 26510 | 208381 ± 65830 | 294508 ± 93038 | 328809 ± 70241 | 170354 ± 10028 | 304372 ± 102852 | 100409 ± 26009 | 64129 ± 4128 |
| HO Sunflower Oil |  |  |  |  |  |  |  |  |  |  |
| (E)-2-Decenal | 984690 ± 236674 | 1194980 ± 246860 | 1097157 ± 260025 | 136694 ± 14305 | 282243 ± 114608 | 3753913 ± 749236 | 4029623 ± 758437 | 864155 ± 277365 | 1004374 ± 326433 | 577736 ± 162073 |
| (E)-2-Octenal | 194032 ± 30670 | 228689 ± 35261 | 306929 ± 42714 | 46180 ± 14474 | 163454 ± 44685 | 250053 ± 48113 | 310181 ± 98274 | 252199 ± 4030 | 327286 ± 44112 | 207102 ± 34775 |
| Decanal | 21774 ± 618 | 20534 ± 3858 | 25215 ± 6246 | 27408 ± 9746 | 58171 ± 12996 | 98474 ± 30375 | 168206 ± 15173 | 184658 ± 6896 | 141952 ± 26101 | 127798 ± 20971 |
| Nonanal | 33516467 ± 2210001 | 20091557 ± 1194482 | 1980983 ± 143659 | 91444984 ± 5021449 | 55036585 ± 6233288 | 198352014 ± 34889347 | 127927303 ± 46111942 | 212563941 ± 57604708 | 38844367 ± 13791054 | 1166752 ± 231386 |
| Linseed Oil |  |  |  |  |  |  |  |  |  |  |
| Nonanoic acid | 99774 ± 14474 | 87449 ± 15666 | 53914 ± 17960 | 6928 ± 4641 | 18380 ± 8046 | 96436 ± 39258 | 64525 ± 11102 | 35203 ± 4371 | 81182 ± 9584 | 40426 ± 1254 |
| 1-Octen-3-ol | 133082 ± 17322 | 133453 ± 18208 | 130138 ± 17930 | 53714 ± 12078 | 89476 ± 12244 | 149369 ± 35314 | 290474 ± 32510 | 781601 ± 26922 | 971075 ± 170854 | 970923 ± 32799 |
| 2-Octen-1-ol | 146866 ± 5929 | 159203 ± 32134 | 154656 ± 67436 | 141493 ± 31595 | 1412236 ± 271360 | 1074984 ± 244886 | 342412 ± 91487 | 478300 ± 52793 | 224861 ± 66097 | 256518 ± 3265 |
| 2.4-Decadienal | 10268 ± 1801 | 13589 ± 2488 | 14712 ± 3978 | 13572 ± 5102 | 21594 ± 8512 | 21362 ± 5555 | 26474 ± 6709 | 59432 ± 8585 | 62852 ± 13855 | 64040 ± 2507 |
| 2.4-Heptadienal | 144562 ± 21778 | 84591 ± 14606 | 18900 ± 5938 | 75255 ± 21836 | 68277 ± 27648 | 582421 ± 187700 | 158296 ± 38491 | 116072 ± 14259 | 191526 ± 53826 | 101324 ± 11529 |
| Heptanal | 961528 ± 371743 | 935317 ± 540021 | 1147416 ± 643333 | 611530 ± 239966 | 565895 ± 116835 | 1452434 ± 624628 | 1406460 ± 637511 | 1426213 ± 445918 | 4249249 ± 1195684 | 415021 ± 20078 |
| 2-Pentyl-furan | 17422 ± 4251 | 21618 ± 5302 | 28427 ± 6737 | 68745 ± 4606 | 184641 ± 51884 | 247764 ± 51783 | 240170 ± 35545 | 242470 ± 56011 | 130693 ± 27576 | 156757 ± 49402 |
| 5-Ethyl-2(5)-furanone | 1689324 ± 445540 | 1752438 ± 601283 | 2275468 ± 661795 | 1708367 ± 626282 | 1407020 ± 562441 | 6542291 ± 2072709 | 19729256 ± 8675076 | 36163819 ± 6134901 | 50346973 ± 14605288 | 39397082 ± 4223269 |
| Hemseed Oil |  |  |  |  |  |  |  |  |  |  |
| Nonanoic acid | 1223218 ± 397619 | 1096914 ± 333127 | 817554 ± 201530 | 127040 ± 27794 | 136284 ± 36238 | 2149909 ± 230040 | 3848733 ± 592128 | 933212 ± 72923 | 1020706 ± 378182 | 642643 ± 55333 |
| 1-Octen-3-ol | 314657 ± 70980 | 395655 ± 69797 | 507056 ± 58995 | 754834 ± 306365 | 1111533 ± 77373 | 2728044 ± 141858 | 1859121 ± 259785 | 2631234 ± 334258 | 1968038 ± 926422 | 2185631 ± 67820 |
| (E)-2-Decenal | 371708 ± 80150 | 426626 ± 61046 | 448727 ± 49458 | 70076 ± 8204 | 193896 ± 151669 | 488384 ± 48838 | 709795 ± 57153 | 356213 ± 39430 | 681846 ± 19126 | 1101604 ± 37214 |
| (E)-2-Heptenal | 9183307 ± 1402293 | 5474476 ± 871949 | 1943676 ± 431459 | 53952709 ± 15757553 | 195677798 ± 43002544 | 436919646 ± 65101027 | 281337879 ± 5309877 | 400937516 ± 43054078 | 407503742 ± 53609309 | 38834800 ± 494247 |
| (E)-2-Nonenal | 150262 ± 72968 | 93692 ± 44320 | 53860 ± 16387 | 6114 ± 1597 | 14622 ± 5013 | 91697 ± 9628 | 51744 ± 16181 | 41077 ± 2120 | 65154 ± 7202 | 46628 ± 1826 |
| (E)-2-Octenal | 170400 ± 30246 | 154604 ± 41157 | 189982 ± 43578 | 121801 ± 36965 | 238603 ± 443 | 548294 ± 59764 | 1092007 ± 260655 | 1566335 ± 506221 | 1794963 ± 734016 | 3258130 ± 926954 |
| 2,4-Decadienal | 484706 ± 110807 | 399039 ± 107078 | 419121 ± 118975 | 857535 ± 41610 | 1402420 ± 455836 | 1797753 ± 348764 | 1703540 ± 254131 | 1672378 ± 637558 | 1414218 ± 212876 | 1886161 ± 214613 |
| 2,4-Heptadienal | 894444 ± 199295 | 826920 ± 147307 | 651201 ± 67323 | 926561 ± 160663 | 918660 ± 66048 | 1584736 ± 337549 | 1037978 ± 161514 | 2055061 ± 253698 | 3415795 ± 90325 | 4234906 ± 871328 |
| Heptanal | 1535749 ± 331038 | 1668719 ± 363260 | 1791707 ± 355006 | 1842899 ± 390552 | 1433366 ± 333272 | 4607247 ± 552451 | 10238327 ± 1462851 | 14333658 ± 2202213 | 17917072 ± 3475795 | 20476654 ± 2195046 |
| 2-Pentyl-furan | 285695 ± 70022 | 164655 ± 36489 | 36841 ± 7696 | 282912 ± 64017 | 159900 ± 34937 | 1946269 ± 275559 | 1615270 ± 229313 | 2368826 ± 395471 | 2151979 ± 414126 | 2302817 ± 213080 |
| 5-Ethyl-2(5)-furanone | 20164 ± 4214 | 27614 ± 4064 | 29367 ± 5292 | 87909 ± 14309 | 83738 ± 22107 | 121564 ± 18127 | 187780 ± 32032 | 157448 ± 21776 | 188319 ± 35786 | 244933 ± 39209 |
